# Supplementary material for: Multivariate analysis of variegated expression in Neurons: A strategy for unbiased localization of gene function to candidate brain regions in larval zebrafish
Source: PLoS One. 2023 Feb 14;18(2):e0281609. doi: 10.1371/journal.pone.0281609 (PMC9928119; doi:10.1371/journal.pone.0281609)
Supplement: S1 Protocol — (PDF) [file pone.0281609.s001.pdf]

Multivariate Analysis of Variegated Expression in Neurons

1 Works for me

h.m.shoen

PROTOCOL INFO

h.m.shoen: Multivariate Analysis of Variegated Expression in Neurons. **protocols.io**
https://protocols.io/view/multivariate-analysis-of-variegated-expression-in-b3p7qmrn

CREATED

Jan 11, 2022

LAST MODIFIED

Oct 10, 2022

PROTOCOL INTEGER ID

56799

GUIDELINES

Do not allow larvae to become dried out at any point.
Be careful when pipetting larvae that have been stored in PBS (not PBS-T)-- they can be very sticky. Using a glass pipette is recommended.
Larvae after bleaching may be difficult to see. It is safe to stop bleaching before the eyes are completely transparent, which can be helpful for spotting them. Otherwise, be sure to work carefully and slowly under excellent lighting conditions to avoid losing larvae.

1 Perform the mating cross for your experiment. Here, we describe an example cross for Gal4 x UAS-induced rescue of the loss-of-function phenotype. Depending on your strategy, your cross may differ. Cross fish that are heterozygous (or homozygous mutant, if available) for a loss-of-function mutation in the gene of interest. At least one fish should carry a Gal4 for the cell type of interest, and at least one should carry a UAS construct that expresses a tagged version of the target gene.

> We kept larvae from different individual mating crosses separate during raising, behavior, and analysis, in case patterns of variegation were substantially different between our mating pairs. Ultimately, when we did not observe major differences between pairs, we analyzed all the data from our different mating pairs together.

Assay phenotype of interest

2

Assay your phenotype of interest in your larvae (See Figure 2 of the associated paper). Sort the larvae according to their phenotype and keep larvae with different phenotypes separate.

> Age of larvae:
>
> While 6 dpf is preferable for registration, we have also successfully registered the brains of 5 dpf larvae. We have not attempted registering brains at any other age. If you assay phenotypes earlier, we recommend allowing larvae to develop to 6 dpf before fixing them. However, this assumes that Gal4 x UAS expression patterns at 6 dpf will reliably report expression patterns earlier in development. Because this assumption may not hold, we strongly recommend finding a way to measure phenotypes as close to 6 dpf as possible.

> Separating larvae into two phenotypic groups:
>
> In our case, phenotypes did not naturally separate into a bimodal distribution for easy division into two groups.

Instead, phenotypes spread along a wide continuum. Larvae exhibited decision-making bias all the way from 100% reoreintation-biased to 100% escape-biased and at every point in between. We chose to collect only larvae on the relatively extreme ends of the spectrum for our analysis. For the escape-biased group, we collected larvae that performed 75% or greater escapes, and for the reorientation-biased group, we collected larvae that performed 75% or greater reorientations. While this somewhat limited our throughput because we discarded a substantial fraction of our larvae, it allowed us to collect two groups with a major difference in their behavior. You will have to use your own judgement to define the groups that you will use for comparison.

Precaution on gain-of-function overexpression-based strategies:

It is important to note that gain-of-function phenotypes can result from expressing a gene at the wrong developmental time or cell type or in unusual abundance. As such, overexpression-induced gain-of-function phenotypes may not necessarily be informative as to the gene's endogenous function. For our CaSR example, we were confident that our gain-of-function phenotype was related to the decision-making function of CaSR for several reasons. First, it was qualitatively the opposite of the loss-of-function phenotype. Second, it was induced by the same manipulation (overexpression in neurons) that rescued the loss-of-function phenotype. Third, it was the same as the effect of applying a pharmacological agonist of CaSR to wild-type larvae (Jain et al. 2018), which naturally express CaSR in the endogenous pattern. Moreover, we confirmed our general results obtained using the gain-of-function strategy with loss-of-function rescue data at every step of our analysis and validation. In summary, the larva collection strategy used for MAVEN should be carefully considered. The gain-of-function strategy was available to us because CaSR exerts bidirectional control of our phenotype of interest. It is likely that most who wish to apply this technique will be best served by the loss-of-function rescue strategy.

3 **Storage of Larve (Optional)** 1d

After phenotyping but before fixation, larvae can be stored in individual wells in 100% methanol for 1-2 days while phenotypic analysis steps are completed. It is entirely possible to skip the methanol step and proceed straight to fixation if desired. Larvae should not be allowed to dry out at any point.

4 **Labeling larval phenotype** 1h

Larvae should be physically labeled such that their phenotype is clearly identifiable (e.g. by cutting their tails bluntly vs. at an angle, pulling off pec fins, etc. We have not attempted removing an eye because we were concerned this would affect registration to the 3D atlas).

All larvae from all phenotypes should be fixed at the same time and stained in the same tube to minimize artifacts.

5 **Fixation** 15m

Fix larvae in 4% PFA in PBS + 0.25% Triton (PBS-T), overnight (O/N) at 4ºC. Note that with MAP-mapping, quick fixation is essential due to the rapid kinematics of ERK phosphorylation, whereas since this protocol only uses tERK, exact timing of fixation is not essential for success. We have found it best to use room-temperature PFA and fix at RT, NOT on ice, then move the larvae to 4 degrees after about 5-10 minutes.

5.1 Wash off PFA with 3 5-minute PBS-T washes.

5.2 Larvae can be stored for 1-2 weeks at 4 degrees in PBS (not PBS-T), or you may proceed directly to "preparation for immunostaining" section.

Preparation for Immunostaining 2h

6 **Bleach larvae** (skip this step if you raised larvae in PTU-- this is only to bleach pigment cells so brains can be imaged without obstruction). After this point, be very careful not to lose larvae in pipetting and washing steps. You may wish to use a glass pipette rather than plastic both for better visibility and to avoid larvae sticking to the sides. 25m

Original PTU protocol:

Karlsson J, von Hofsten J, Olsson PE (2001). Generating transparent zebrafish: a refined method to improve detection of gene expression during embryonic development.. Marine biotechnology (New York, N.Y.).

Note on PTU: We strongly encourage validating that the phenotype of interest is not affected by PTU before using it on larvae for this protocol. PTU is known to affect autophagy, eye development, and visual behaviors. If there is any doubt, it is better to raise larvae in normal embryo media and bleach them after fixation.

PTU reduces eye size:

Li Z, Ptak D, Zhang L, Walls EK, Zhong W, Leung YF (2012). Phenylthiourea specifically reduces zebrafish eye size.. PloS one. https://doi.org/10.1371/journal.pone.0040132

PTU alters visual behaviors:

Antinucci P, Hindges R (2016). A crystal-clear zebrafish for in vivo imaging.. Scientific reports. https://doi.org/10.1038/srep29490

PTU induces autophagy:

Chen XK, Kwan JS, Chang RC, Ma AC (2021). 1-phenyl 2-thiourea (PTU) activates autophagy in zebrafish embryos.. Autophagy. https://doi.org/10.1080/15548627.2020.1755119

6.1 Prepare bleaching solution fresh every time: for 1 mL, combine 700 uL PBS-T, 200 uL 5% KOH, and 100 uL 30% H2O2. 5m

Hydrogen Peroxide **Sigma Aldrich Catalog #H1009-500ML**

6.2 Incubate on a rocker at RT for about 10 minutes or 55 degrees for about 5 minutes, until eyes are light orange in color, then rinse the bleach immediately off with 2-3 quick PBS-T washes, followed by one 5 minute PBS-T wash. Larvae will continue to bleach until they are completely washed, so be careful to begin washing when they are slightly darker than needed. 20m

7 Antigen retrieval 35m

Some MAP-mapping protocols say this step can be skipped. We find that it is critical for proper tERK signal and do NOT recommend skipping it.

7.1 Incubate in 150mM TrisHCl (pH 9) for 5min at RT 5m

7.2 Transfer to 70°C water bath for 15min 15m

7.3 Wash with PBS-T-- one quick rinse, then 2x 5 min 10m

8 **Permeabilize larvae** 1h

Thaw trypsin 0.05% Trypsin -EDTA on ice.

Trypsin-EDTA 0.05% phenol red **Gibco - Thermo Fischer Catalog #25300054**

8.1 For 6 dpf larvae, incubate in trypsin on ice for 45 minutes 45m

For all MAVEN experiments, the trypsin incubation step was 45 minutes. Others in our lab have found that 10 minutes in trypsin on ice is sufficient for antibody penetration in 5-6 dpf larvae, and that the shorter incubation saves time and better preserves structural integrity of larvae.

8.2 Rinse the trypsin off with two quick washes in PBS-T, visually confirming that all the pink is gone, then wash for 10 min with PBS-T. 15m

Immunostaining 2d 0h 45m

9 **Block larvae**

9.1 Make blocking solution in PBS-T (can make in large batches and freeze aliquots):
2% Normal Goat Serum (only use NGS if no primary antibodies are made in goat or sheep)
1% BSA
1% DMSO

9.2 Incubate for 1 hour on a rocker at room temperature. In a 1.5 mL Eppendorf tube, use ~1 mL of blocking solution. Do not re-use blocking solution.

10 **Apply primary antibody**

10.1 Dilute antibodies 1:200 for anti-GFP and 1:500 for anti-tERK in 1%BSA, 1%DMSO in PBS-T 5m

chicken anti-GFP **ROCKLAND antibodes & assays Catalog #600-901-B12**

anti-tERK **Cell Signaling Technology Catalog #4696**

Note: this protocol should hypothetically work with any tagged expression construct, not just GFP-tagged constructs. Substitute antibodies as appropriate in order to visualize your expression construct.

Remove blocking solution from larvae and apply primary-- I use 1000 uL of primary in a 1.5 mL Eppendorf tube for 50-100 larvae, but less can be used if necessary as long as the larvae remain completely submerged in primary while incubating overnight.

10.2 Incubate overnight on a gentle rocker or rotator at 4 degrees C 12h

10.3 Wash off primary 3X 15 min in PBS-T on rocker at room temperature. 45m

It is possible to save the primary and re-use it, but primary may diminish in concentration and suffer from repeated freeze-thaw cycles over time, so re-use primary antibody at your own risk.

11 **Apply secondary antibody** 12h 50m

11.1 We used **Goat anti-Mouse IgG1 Cross-Adsorbed Secondary Antibody Alexa Fluor™ 633 Thermo Fisher Scientific Catalog #A-21126** to label tERK in red and **Alexa Fluor® 488 AffiniPure Donkey Anti-Chicken IgY (IgG) (H L) Jackson Immunoresearch Catalog #703-545-155** to label GFP. Dilute both antibodies 1:500 in 1% BSA, 1% DMSO in PBS-T, taking care not to expose to light. 5m

11.2 Incubate overnight in the dark (for example, by placing in an opaque box or covering with aluminum foil) on a gentle rocker or rotator at 4 degrees C 12h

11.3 Wash off secondary in the dark 3X 15 min in PBS-T on rocker at room temperature. 45m

12 Store at 4 degrees in total darkness in 2:1 **VECTASHIELD Mounting Medium Vector Laboratories Catalog #H-1000** : PBS mixture for at least overnight or up to 2 weeks. I find that pre-incubating the larvae in a Vectashield:PBS mixture prevents them from shrinking / wrinkling, which occurs when they are moved straight from PBS to pure Vectashield. Generally speaking, many storage and/ or mounting methods are likely acceptable, so long as they preserve the shape of the brain.

Imaging

13 **Imaging**

Before imaging of experimental larvae, it is highly advisable to prepare test larvae and attempt to register brains to the ZBrain registration image using your imaging settings. For example, the original Randlett et al. paper uses a 20X water immersion lens for imaging. We used a 20X air lens at 0.8X digital zoom. We also tested a 10X air lens with 1.6 digital zoom, but were not successful. You may have to modify this section heavily depending on your microscope-- ultimately, the highest priority is to find settings that allow you to reliably register your brains to the reference brain. Consult the reference brain when choosing your own settings. In particular, make sure that the deepest parts of the brain are still visible.

13.1 Mount Vectashield-soaked larvae in 1.1%- 1.25% low-melt agarose in a glass-bottomed petri dish, dorsal side down. Multiple larvae can be mounted in the same dish for higher efficiency.

It is IMPERATIVE that the larvae are mounted with no tilt, either left to right or front to back. Even small amounts of tilt will compromise later registration to the reference brain. When in doubt, it is better to very gently unmount and re-mount a larva than to proceed with a tilted specimen.

13.2 As larvae are mounted, be sure to examine their tails carefully to determine what phenotype they had. Document this information (and their positions in the dish, if you are mounting multiple larvae in

the same dish).

13.3 For our experiments, we used a Zeiss 880 microscope with a 20X 0.8 NA air lens at 0.8 zoom. The "tile" function of Zen was used to capture and stitch together two images, one including the forebrain, midbrain, and rostral part of the hindbrain and the other including the caudal hindbrain and anterior spinal cord. Step size was 2 microns. A brain usually comprised around 130-150 slices. Laser intensity and gain were calibrated such that the brightest neurons in the brain were saturated, because otherwise signal in the dimmest neurons was lost. (Note that saturated pixels exist in some portions of the reference brain as well-- it is best to attempt to match the staining of the reference brain as closely as possible.) 3-5 larvae were inspected before final settings were chosen, due to the variability in brightness of the GFP signal between brains. Ideally, the full range of each channel should be utilized. Once settings were determined, the same imaging settings were used for every brain in a staining batch. Images were saved in 8-bit, because they will be downsampled to 8-bit at a later step anyway.

For a sense of exactly which parts of the fish to image, see the reference brain. This image includes the entire forebrain and olfactory pits all the way back to the pectoral fins. Neglecting to include parts of the brain and spinal cord in your image that are included in the reference brain, or including regions that are not included in the reference brain, can lead to stretching problems with the registration.

LSM880 with Airyscan
Confocal microscope
Zeiss **LSM880**

.

13.4 After imaging, place each individually-identified larva in a well of a genotyping plate, keeping careful track of which larva corresponds to which image and phenotype.

14 Once imaging is complete, genotype the larvae according to your own protocol.

Another option is to genotype larvae before phenotyping and fixing them. To pre-genotype live zebrafish larvae, we recommend the protocol by Zhang et al (2020). If you use this protocol, you must somehow mark which genotype the larvae have, just as you marked which phenotype they have. Note that this prevents you from performing the imaging part of the protocol blind to genotype.

Zhang X, Zhang Z, Zhao Q, Lou X (2020). Rapid and Efficient Live Zebrafish Embryo Genotyping.. Zebrafish.
https://doi.org/10.1089/zeb.2019.1796

When we enriched our samples for mutants, we used their protocol with some modifications, described here. Briefly, 2 dpf larvae were dechorionated by pretreating with pronase. Larvae were rinsed 3X in DNA collection buffer with tricaine, placed in DNA collection solution, and incubated at 37 degrees for 30 minutes without shaking. Supernatant solution was mixed with lysis buffer and incubated at 95 degrees for 5 minutes, while embryos were returned to E3 in individual wells. Supernatant solution was then genotyped using proprietary KASP primers from LGC Genomics. Note that KASP primers often work well with very small amounts of gDNA-- other genotyping protocols, particularly those that require more gDNA, may not succeed.

15 Preparing images for registration in FIJI

FIJI (Image J)
by NIH

If you have not already installed FIJI on your computer, see the installation guide + downloads here:

https://imagej.net/software/fiji/downloads

15.1 Orient brains exactly vertically in FIJI using Transform Rotate. Even slight deviations from perfectly vertical can cause registration errors -- even a 2% rotation is worth it.

15.2 Split channels using Colors Split channels

15.3 If you imaged on the Zeiss 880 with the dorsal side of the fish closest to the coverslip, you must flip Z orientation using Transform Flip Z (as stack number goes higher, you must approach the dorsal side of the brain—look at the reference brain to be sure you've got it right. If your brain is not in the same orientation as the reference brain, the registration will fail.)

15.4 Save individual channels as .nrrd files with _01 suffix for the tERK channel and _02 for the GFP (or other marker of your expression construct) channel.

Registration to the reference brain

16 Registration to reference brain

> CMTK Registration Runner was developed by Sándor Kovács. CMTK was developed by Torsten Rohlfing. Munger was developed by Greg Jefferis. Parameters for registering zebrafish brains to reference brain were determined by Owen Randlett. Reference zebrafish brain image was taken by Owen Randlett and is hosted on FishExplorer, a website maintained by the Engert lab.

16.1 For alternative instructions using the command line, see Randlett et al. (2015)

In order to facilitate this step for those who are not comfortable using the command line, we strongly recommend using the CMTK Registration Runner GUI by Sándor Kovács. There are detailed instructions to install and use this program at the link below. Install the version appropriate for your operating system.

https://github.com/sandorbx/Fiji-CMTK-registration-runner-GUI#readme

16.2 Once installation is finished, register brains. Note that these instructions are for a Windows user; Mac / Linux users will need to modify. Begin by opening MobaXterm, the Linux emulator you just installed from the link above.

16.3 In the left side menu, click WSL-Ubuntu-20.04. This should open a new tab in MobaXterm with the header "WSL-Ubunut-20.04." In that tab, type pcmanfm and press enter.

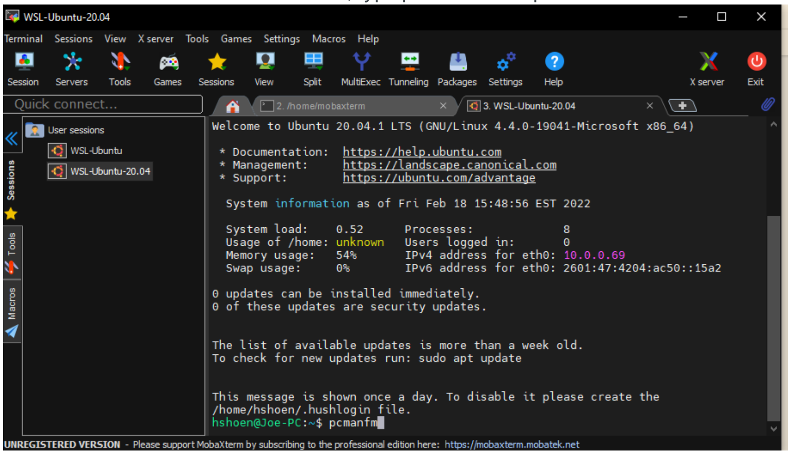

A screenshot of what MobaXterm will look like just before pressing "enter."

16.4 A new window will appear. Click on Fiji.app and then click on ImageJ. In the new dialog box that appears, click on "Execute." You *don't* need to click "Execute in Terminal" even though this is what you are prompted to click.

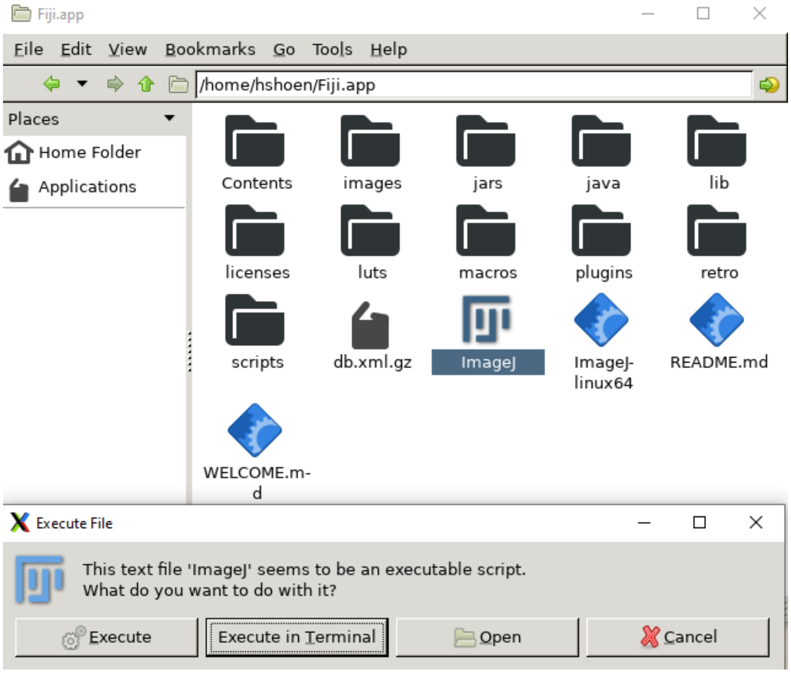

A screenshot of how to open FIJI in MobaXterm

16.5 Open CMTK Registration Runner by opening the "plugins" menu, then the "macros" submenu, then clicking on "Fiji CMTK registration runner."

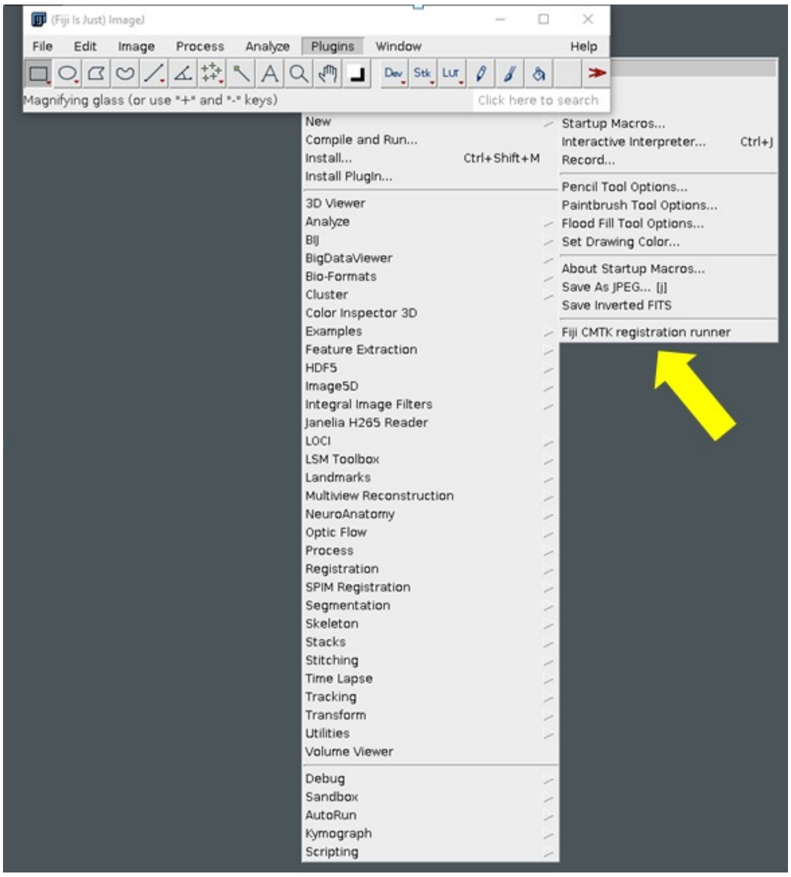

How to open FIJI CMTK Registration Runner in ImageJ. Yellow arrow indicates where to click.

16.6 You will need to download the reference brain from the ZBrain 2.0 atlas before you can register your

brains. To do this, go to this link: https://zebrafishatlas.zib.de/downloads. On the right side, "Other", there will be a button for "Reference brain." Move the reference brain file to an easy-to-access place in your file structure, but do not put it in the same folder as the brains that you will be registering.

16.7 For the field "CMTK library with Munger" navigate to the file Fiji.app/lib/cmtk_munger_wsl_linux and click "Select." You will only have to do this once.

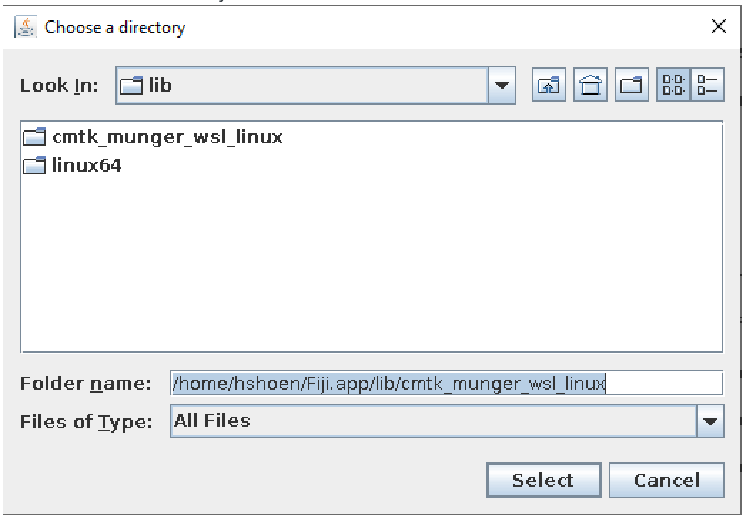

Navigating to Fiji.app/lib/cmtk_munger_wsl_linux

16.8 For "reference brain (file)" navigate to the reference brain image file.

16.9 For "images to register (directory)" navigate to the folder in which all of your brain images have been saved. They should be .nrrds with the suffix _01 for the tERK file and _02 for the GFP file.

For "output selection" we recommend making a new folder for your registered brains.

16.10 The image registration parameters, taken from Randlett et al. 2015, are as follows:

- awr 010203 –T -8 –X 52 –C 8 –G 80 –R 3 –A ‘accuracy 0.4’ –W ‘accuracy 1.6’

In CMTK Registration Runner, this translates to:

- a run affine transformation - CHECK
- w run warp transformation - CHECK
- c channels for registration - CHECK the number of channels in your images
- r run reformat on those channels - CHECK
- T (threads) default auto - Number of compute threads to use -- user's choice, depends on computer's capabilities
- X (exploration) 52
- C (coarsest) 8
- R (refine) 3
- G (grid spacing) 80
- Accuracy 1.0

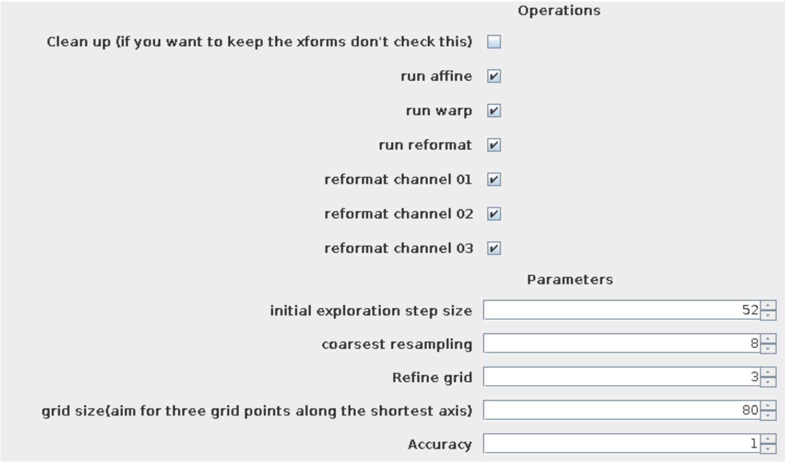

A screenshot of parameters entered into CMTK Registration Runner

Click "OK" to run your registration. This may take some time (e.g. hours), depending on how many brains you are registering and how many threads you are using.

16.11 Once the brains are done registering, open the registered brains in Image J along with the reference brain. Carefully compare the tERK channel in the registered brain with the reference brain. If the brain has not registered correctly, there are two options. The first is to exclude it from the analysis. The second option is to take all of the incorrectly-registered brains from a single batch and register them to a brain from the same batch that did register correctly, as an intermediate step, then re-register all of those brains to the reference brain. This sometimes succeeds if the tERK staining pattern varies only slightly from the reference brain's, likely due to batch effects in staining. If the fish were poorly positioned during imaging, it is unlikely they can be saved.

16.12 Make sure your registered brains are in their own folder with no other files or subfolders in it. At this point, you can set aside the registered tERK channels (with the suffix _01). All future steps are for the GFP channels (with the suffix _02) only.

Smooth and reformat your registered brains using the PrepareStacksForMAPMapping.ijm macro in FIJI. This macro can be found at Owen Randeltt's github page, https://github.com/owenrandlett/Z-Brain, or here: **PrepareStacksForMAPMapping.ijm** . Make sure that the maximum pixel intensity ("max =" __, line 3) is correct-- if you're using 8-bit images, the correct number is 256. Once you click "Run" you will be asked to direct the computer to the file containing your .nrrd output images as well as a new folder where the smoothed and reformatted images should go. Running this step should be substantially faster than registration (e.g. 15 minutes or less).

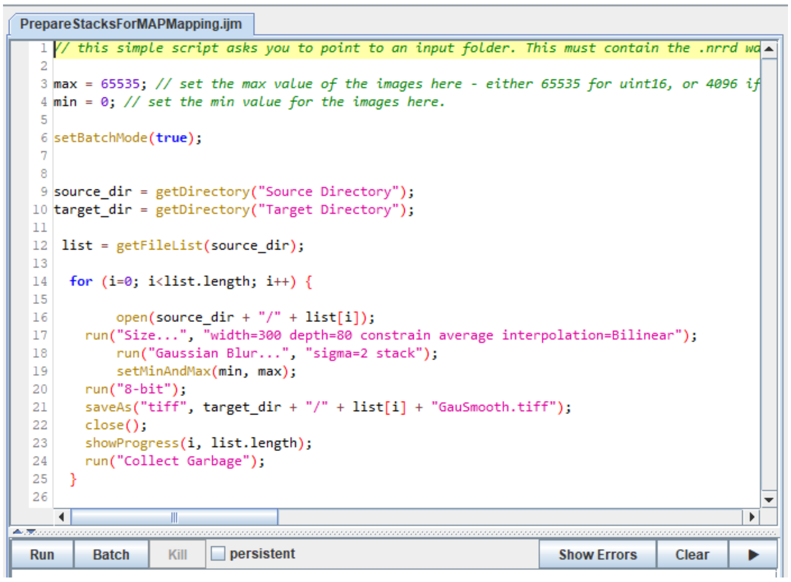

The ImageJ macro "PrepareStacksforMAPMapping.ijm"

After this step, in your output folder there should be a new .tiff file corresponding to each of your registered .nrrd files. If you've allowed the default naming of each step, your filenames should be something like "Ref20131120pt14pl2_Fish1_02_warp_m0g80c8e1e-1x52r3.nrrdGauSmooth.tiff"

Quantification of signal in each brain region

17 This step, quantification of GFP signal in each brain region, requires the use of Matlab. 1h
https://www.mathworks.com/products/matlab.html

You will need to download the file 'AnatomyLabelDatabaseDownsampled.hdf5' from https://zebrafishatlas.zib.de/downloads (under the "Others" header at the far right).

You will need to download the file 'MaskDatabaseDownsampled.hdf5' here:
MaskDatabaseDownsampled.mat

Since the Matlab section of this code will likely only take <1 hour to run, assuming you have gathered all the necessary information in advance, it may be possible to run on a shared computer or using the Matlab free trial, if purchasing the program is not an option.

The code in this section was modified from code originally written by Owen Randlett.

Randlett O, Wee CL, Naumann EA, Nnaemeka O, Schoppik D, Fitzgerald JE, Portugues R, Lacoste AM, Riegler C, Engert F, Schier AF (2015). Whole-brain activity mapping onto a zebrafish brain atlas.. Nature methods.

17.1 The function QuantifySignalMultipleBrains.m will take as an argument your chosen output file name. Within the function, you will point Matlab to a folder containing the .tiff files of your aligned, smoothed brains. The function also requires the files 'AnatomyLabelDatabaseDownsampled.hdf5' and "MaskDatabaseDownsampled" to on the computer, and you will be asked to direct Matlab to the folder containing these files.

For those new to Matlab -- your command will look like this: QuantifySignalMultipleBrains("YourDesiredFileNameHere"). Don't forget the quotes! There is no need to have a ".csv" in the filename.

The function will loop through all of your .tiff files, asking you to input values for key variables that will go into the column name corresponding to that brain. The output of the function will be a .csv file. Each column of the file corresponds to a single brain. Each row corresponds to the signal intensity in one of the 293 brain regions in the anatomy database.

\Each column header contains some metadata about the fish that you provided.

If a given piece of metadata is not provided, its space is filled with an "x". If the metadata is out of order or not marked by an "x" the R code in the next step will not function as intended.

	A	B
1	ROIname,	LLC16_het_LLC_210418_210510
2	Diencephalon -	12.3597
3	Diencephalon - Anterior group of the posterior t	3.9826
4	Diencephalon - Anterior preoptic dopaminergic	4.4881
5	Diencephalon - Anterior pretectum cluster of vn	13.4112
6	Diencephalon - Caudal Hypothalamus	2.0979
7	Diencephalon - Diffuse Nucleus of the Intermed	19.6937
8	Diencephalon - Dopaminergic Cluster 1 - ventral	6.237
9	Diencephalon - Dopaminergic Cluster 2 - posteri	6.3321
10	Diencephalon - Dopaminergic Cluster 3 - hypoth	1.7845
11	Diencephalon - Dopaminergic Cluster 4/5 - poste	3.1763
12	Diencephalon - Dopaminergic Cluster 6 - hypoth	3.1653

Screenshot from Excel showing example output from the QuantifySignalMultipleBrains function. In the left column, "ROIname," (Region of Interest Name) are the names of brain regions from the anatomy database. In the right column, we have a brain name that describes, from left to right, the filename, the genotype, the phenotype ("LLC" refers to a fish that performed predominantly long-latency C-bends on a decision-making assay), the date the fish was stained, and the date the fish was imaged. Values in the cells are raw GFP signal intensity.

If you enter anything incorrectly while the Matlab program is looping, you can always make a note of it and fix the mistake in the column headers later. It is important not to have any typos in these headers, as they will be used as variables for analysis once the data is imported into R.

During this step, you may choose to modify the information collected about each brain by editing the Matlab code (for example, if you are working with a double mutant, you will need to add a step where you document the genotype for Gene A and for Gene B). Keep in mind that you will have to tweak the file import and data processing in R if you choose to do this.

Import data into R 1h

18 This section requires RStudio, which is open source and free.

For your own analysis, you may either choose to modify the example analysis that we present here, or create your own R script or RMarkdown document based upon these steps and our RMarkdown example. Typically an R script will be simpler for a new user of R to work with, but an RMarkdown document can be used to generate reports in html or .pdf format. You can always start by creating an R script, then adapting the code into an RMarkdown format once it is working.

For new R users, we recommend referring to the book **R for Data Science** by Hadley Wickham and Garret Grolemund. It is available free as an ebook (with author permission) here.
If a hard copy is desired, one can be purchased here.

For those interested in learning more about multivariate analysis and its implementation in R, we recommend referring to **An Introduction to Statistical Learning**. The .pdf is available for download (with author permission) here. If a hard copy is desired, one can be purchased here. StatQuest (https://statquest.org/) is also an excellent free online resource for those with little background in multivariate statistics.

18.1 I have provided an example analysis in the following RMarkdown files.

MAVEN_Whole_Project_220222.Rmd **MAVEN_Whole_Project_220222.html**

For examples of data analysis, see the RMarkdown files (html file is an interactive "tour" of analysis and figures, while RMarkdown file contains editable code for performing all the analyses and generating all the figures in the .html file).

Before you can analyze your own data, you will need to install packages, load some custom functions (attached below) and modify the data import section of this code. Instructions for each step are provided below.

Some basic tips for working with RMarkdown:

- Press CTRL + SHIFT + ENTER to run a code chunk
- Press CTRL + ENTER to run a single line of code
- Use the little triangles on the far left (next to the line numbers) to collapse code you're not working with
- Working with entire chunks of code can be unwieldy. If you need to troubleshoot a piece of code, it's often a good idea to copy and paste it into a new script and work with it there, then put the fixed code back in RMarkdown
- RMarkdown has different rules for default working directories than R, so double check your file paths if you're having trouble

18.2 R allows users to develop "packages" which contain specialized user-created functions. I employ several packages in my analysis. One option is to install the following packages manually in R using the Packages--> Install menu in the lower right corner of RStudio. Once a package is installed, it must be loaded using the library() command. 1h

- tidyverse
- glmnet
- readxl
- janitor
- hablar
- ggpubr
- here
- gt
- ComplexHeatmap
- circlize
- corrplot
- renv

Alternatively, to run the code with exactly the same versions of these packages that I used:
1) Install the renv package
2) Make sure the renv.lock file is in your working directory renv.lock
3) Run the command renv::restore()

18.3 Next, following the R code, we load some custom functions and associated files for the analysis. Download these files and place them in the same folder as the R Project you're working with. More details on what these functions do are available in the comments of the R code (step 19.5).

export_top_PC_regions.R exportStrongestCorrelatedRegions.R filterCaudalBrain.R graph_PC_components.R normalizeSignalBy.R tidyImportedDataUnderscore.R 251_BrainRegions.xlsx 275_BrainRegions.xlsx 293_brain_regions_formatted.csv

Example code to load a function:
source("exportStrongestCorrelatedRegions.R")

18.4 Next, following the R code, we load into R the raw brain region signal intensity data that we generated using Matlab.

We assume that each sample name contains some metadata about the fish: namely, its number, the pair it came from, its genotype, phenotype, the date it was collected (e.g. the date behavior was performed) and the date it was imaged. Thus, the FishName column contains entries that look like this:

Fish10_41_mut_LLC_210314_210320

In the Matlab code, if a given piece of metadata is not provided by the user, its space is filled with an "x". If the metadata is out of order or not marked by an "x" the R code will not function as intended.

You will need to modify the code to point it to your Matlab output file.

The custom function "tidyImportedDataUnderscore.R" contains the code that transposes the imported file and splits the long FishName into the columns "FishNum," "pair," "geno," "pheno," "collected," and "Imaged." If you altered the Matlab code for generating descriptive column headers for larvae, you should modify lines 23-29 of the tidyImportedDataUnderscore function accordingly. Each unique descriptor of your data should get its own column.

	unique_id	fish_num	pair	geno	pheno	collected	imaged	diencephalon
1	LLCF01_55_het_LLC_210123_210213	LLCF01	55	het	LLC	210123	210213	0.43993000
2	LLCF03_55_het_LLC_210123_210213	LLCF03	55	het	LLC	210123	210213	0.49737000
3	LLCF04_55_het_LLC_210123_210213	LLCF04	55	het	LLC	210123	210213	1.439500
4	LLCF05_55_het_LLC_210123_210213	LLCF05	55	het	LLC	210123	210213	1.123800
5	LLCF06_58_het_LLC_210124_210131	LLCF06	58	het	LLC	210124	210131	2.09480000
6	LLCF09_58_het_LLC_210124_210131	LLCF09	58	het	LLC	210124	210131	0.553060
7	LLCF10_58_het_LLC_210124_210131	LLCF10	58	het	LLC	210124	210131	0.5393900
8	LLCF11_58_het_LLC_210124_210131	LLCF11	58	het	LLC	210124	210131	0.1717200
9	LLCF01_58_het_LLC_210124_210131	LLCF01	58	het	LLC	210124	210131	0
10	LLCF02_58_het_LLC_210124_210131	LLCF02	58	het	LLC	210124	210131	0.5585400
11	LLCF03_58_het_LLC_210124_210131	LLCF03	58	het	LLC	210124	210131	1.9265000
12	LLCF04_58_het_LLC_210124_210131	LLCF04	58	het	LLC	210124	210131	0.75531000

RStudio console showing data after the tidyImportedDataUnderscore function has been applied. Besides transposing the data, this function also breaks up the long fish description into individual variables, which can now be grouped by and sorted for. If alterations were made to experimental design earlier in the pipeline, this function must also be modified.

18.5 We also load in a list of the names of all 293 brain regions plus corresponding abbreviations for these brain regions. We often use the abbreviated forms in our graphs because some of the true anatomical brain regions are long enough to interfere with axis labels for figures. If you want to move between different forms of brain region names, you can use a join function to combine your data with this key, then the dplyr select function to retain only the form of the names that you want to work with.

293_BrainRegions_Translator.xlsx

18.6 In the code **Section 7.1 Correlational analysis: which other regions correlate with the DCR6?** of the R code the user generates a list of brain regions where signal is most highly correlated with another brain region. This can be used to identify alternative candidate phenotype-causative regions if the first region identified by LASSO regression fails to validate. The R code exports a .csv file in a format that can be read by the Matlab function **CustomBrainRegionStack.m**, which can be used to generate customized images showing the anatomical arrangement of the set of brain regions specified. A detailed description of how to use that function is contained in its header.

18.7 From this point forward, use the comments and instructions within the RMarkdown code chunks to guide your own analysis. After every step, be sure to stop and think about the results and what they imply for future steps. It is likely that every analysis will be slightly different, since the underlying structure of the variation in gene expression will be slightly different. This code should provide a useful framework, but a good grasp on multivariate statistics is also essential to help interpret results as you go. If you need help, refer to resources in the note of Step 18. Good luck!
