# Supplementary material for: Multivariate analysis of variegated expression in Neurons: A strategy for unbiased localization of gene function to candidate brain regions in larval zebrafish
Source: PLoS One. 2023 Feb 14;18(2):e0281609. doi: 10.1371/journal.pone.0281609 (PMC9928119; doi:10.1371/journal.pone.0281609)
Supplement: S1 Code — (ZIP) [file pone.0281609.s003.zip › CODE/MAVEN_Whole_Project_221106.html]

Walking through MAVEN and associated analyses


Code 

- Show All Code
- Hide All Code

# Walking through MAVEN and associated analyses

#### Hannah Shoenhard

#### 2/24/2022

# 1 Introduction

This code is the final part of an analysis pipeline for whole-brain expression data in zebrafish. Briefly, the purpose of this analysis is to identify individual brain regions where overexpression levels of a tagged gene of interest are correlated with a phenotype of interest. The analysis here is based on the following:

- Mutations in the Calcium-sensing Receptor (CaSR) gene lead to a decision-making phenotype in which acoustic stimuli largely elicit LLCs (reorientations) instead of the typical SLCs (escapes).
- Overexpressing CaSR in neurons can rescue the CaSR mutant LLC-biased phenotype. It also drives CaSR siblings (that is, WT and heterozygous larvae) to be even more SLC-biased than usual.
- We noticed that the Gal4 x UAS system we used to drive CaSR-EGFP expression in neurons produced variegated expression
- We determined that larvae expressing more CaSR-EGFP (as measured by approximate brightness of the GFP signal) were more highly shifted towards SLCs. However, sometimes even bright larvae were not shifted.
- Therefore, we hypothesized that CaSR-EGFP expression levels in a *specific* brain region drive the shift towards SLCs
- If this is the case, then CaSR-EGFP expression levels in the relevant brain region will be the most predictive of phenotype, out of all brain regions

We therefore set out to develop an analysis pipeline that could identify candidate brain regions where CaSR-EGFP expression may drive shifts in phenotype towards SLCs. By this point in the pipeline, brains of larvae with and without the phenotype of interest have been aligned to a reference brain in ImageJ using CMTK Registration Runner. Next, signal from the tagged gene of interest has been quantified in anatomically-defined brain regions using Matlab. Instructions for both of these steps are available on protocols.io Now, this document will walk you through the multivariate analysis, including:

- data import
- data wrangling
- exploring your data using heatmaps and correlations (Figure 3)
- exploring your data using PCA
- choosing whether and how to normalize your data
- LASSO regression to identify brain regions where signal most strongly predicts phenotype (Figure 4)
- Two-way ANOVA to confirm LASSO regression results (Figure 4)
- Bootstrapping logistic regressions to assess relative predictive ability of signal in your brain region vs. average signal (Figure 4)
- Methods to identify alternative candidate brain regions if experimental validation of the original candidate region fails (Figure 6)

It is important to note that this analysis is correlative. Once a candidate is identified using this method, it must be experimentally validated in order to show causation. We used ZBrain 2.0 to identify other Gal4 lines that drove expression semi-specifically in our region of interest, then demonstrated that CaSR-EGFP expression driven by these lines alone was sufficient to shift larvae towards SLCs.

We gratefully acknowledge the DIY Transcriptomics course by Dr. Daniel Beiting of UPenn’s School of Veterinary Medicine for freely providing example code. Scripts and instructional videos can be found at the link above.

Note: by default, all code chunks are hidden in the knit RMarkdown document. To display the code used in a specific chunk, click the “code” button to the right.

Note: The terms “Rhombencephalon QRFP Cluster - Sparse” and “Dorsal Cluster Rhombomere 6” (DCR6) refer to the same brain region. We refer to the region as the DCR6 because we do not believe it truly expresses QRFP (see Chen et al. 2016, “QRFP and its receptors regulate locomotor activity and sleep in zebrafish”).

# 2 R packages used for this analysis

These packages must be installed and loaded.

```
#### load packages ####

# As long as the renv.lock file is available, the exact packages used to run this code can be restored by running the command renv::restore()

library(tidyverse)
library(glmnet)
library(readxl)
library(janitor)
library(hablar)
library(ggpubr)
library(here)
library(gt)
library(ComplexHeatmap)
library(circlize)
library(corrplot)
library(renv)

# Using renv to store packages that were in my environment when I wrote and used this code
#renv::init(bioconductor = TRUE)
#renv::snapshot()
# use renv::restore() to load from the .lock file
```

# 3 Custom functions used in this analysis

These functions must be in the working directory to load properly.

```
source("filterCaudalBrain.R")
source("tidyImportedDataUnderscore.R")
source("normalizeSignalBy.R")
source("graph_PC_components.R")
source("export_top_PC_regions.R")
source("exportStrongestCorrelatedRegions.R")
```

# 4 Importing expression data

Now, we import the quantification of the gene of interest from Matlab. Each row is assumed to be a single brain and each column is assumed to be a single brain region. Cells contain the signal in each brain region for a given fish, which was normalized in Matlab to the size of that region.

We assume that each sample name contains some metadata about the fish: namely, its number, the pair it came from, its genotype, phenotype, the date it was collected (e.g. the date behavior was performed) and the date it was imaged. Thus, the FishName column contains entries that look like this:

```
  Fish10_41_mut_LLC_210314_210320
```

In the Matlab code, if a given piece of metadata is not provided by the user, its space is filled with an “x”. If the metadata is out of order or not marked by an “x” the R code will not function as intended.

```
#This code shows an example of importing data that was analyzed on six different dates and combining it into one object.You could also put all your data into a single spreadsheet and import it as a single object. 

raw_data <- read_csv("raw_expression_data_for_MAVEN.csv")

filteredData <- filterCaudalBrain(raw_data, "rhomb7") #my custom funciton for excluding regions within rhombomere 7 and caudal to it, because my registrations did not properly align these. If your registration performed better, skip this step

tidiedData <- tidyImportedDataUnderscore(filteredData) %>%
  arrange(pheno, geno)
#my custom function for parsing out columns with metadata that was entered into fishname in Matlab
```

## 4.1 Wrangling data

We create a ‘studyDesign’ object to store the metadata about each fish and a `splitColsMatrixNumeric` object to store the numerical data about all the brains. This will be the input for our Principal Component Analysis (PCA) and any other analysis that requires a matrix of expression data only (no character columns).

# 5 Exploratory analyses

## 5.1 Principal Component Analysis

If you are not familiar with PCA, we highly recommend referring to **An Introduction to Statistical Learning with Applications in R** (full citations in main paper). Some code in this section has also been modified from StatQuest’s “PCA in R” video by Dr. Josh Starmer at UNC Chapel Hill.

We see that 90% of variance is explained with the first 9 PCs.

```
matrix <- as.matrix(numericDataOnly) #PCA requires matrix input
rownames(matrix) <- studyDesign$unique_id

pca <- prcomp(matrix, scale=TRUE) 

#peek at data
#summary(pca)

## make a screeplot
pca.var <- pca$sdev^2
pca.var.per <- round(pca.var/sum(pca.var)*100, 1)
screeplot <- barplot(pca.var.per, main="Screeplot", xlab="Principal Component", ylab="Percent Variation")
```

```
#screeplot
```

### 5.1.1 Prepare for graphing

This reintegrates the metadata that I had to remove in order to do PCA analysis, and reshapes the data to be compatible with ggplot.

```
#this formats data the way ggplot likes it 

top_count <- as.numeric(length(studyDesign$fish_num))

pca.data <- data.frame(fish_num = studyDesign$fish_num,
                       pheno=studyDesign$pheno,
                       geno = studyDesign$geno,
                       pair = studyDesign$pair,
                       imaged = studyDesign$imaged,
                       collected = studyDesign$collected,
                       unique_id = studyDesign$unique_id,
                       count = seq(from = 1, to = top_count),
                       PC1=pca$x[,1],
                       PC2=pca$x[,2],
                       PC3=pca$x[,3],
                       PC4=pca$x[,4],
                       PC5=pca$x[,5],
                       PC6=pca$x[,6],
                       PC7=pca$x[,7],
                       PC8=pca$x[,8],
                       PC9=pca$x[,9])

# orders the fish correctly for data display (continue to be sorted by phenotype instead of being ABC'd)
pca.data$unique_id <- factor(pca.data$unique_id, levels = pca.data$unique_id[order(pca.data$count)])
```

### 5.1.2 Small multiples plot: phenotype

Small multiples plots can give us a sense of whether certain variables map onto certain PCs. It doesn’t appear that the phenotype is mapping onto any particular PC.

PCA reduces the dimensionality of the dataset without any information about phenotype. PCs represent correlated patterns of variation in the data (in this case, CaSR-EGFP signal in various brain regions). If we suspect CaSR is acting in a single brain region, we wouldn’t necessarily expect any PC to map directly onto phenotype, unless CaSR-EGFP signal in that brain region happens to be a major contributor to that PC.

### 5.1.3 Small multiples plot: date collected

In my experiments, I put all the fish from a single date into the same tube for staining. I am concerned about tube artifacts, so I want to check if the tube that a fish was stained in affects its whole brain staining pattern. Upon examining the data, it seems that the fish from both January dates are consistently skewed in PC1, which covers 69% of all variance.

### 5.1.4 Average Brightness Analysis on Non-Normalized Data

I wish to further explore the differences between tubes (batch effects) that have been suggested by PCA. Here, I average the signal across each brain, then plot the brains sorted by the date collected / tube / batch. There is a clear difference between dates, especially the two January dates and every other date.

## 5.2 Normalizing data by date collected / tube

I wrote a function that normalizes data within the specified group, and here I apply it to the raw dataset that I used for the analysis above, then re-do the analysis on the normalized data.

### 5.2.1 PCA post-normalization

We now repeat the analysis and see that the batch effects of the January tubes have been partially, but not entirely, mitigated when examined via PCA.

```
ggplot(pca_normed.pivot) +
  aes(x=unique_id, y=loadings, fill=collected) + 
  geom_bar(stat="identity") +
  facet_wrap(~PrincipalComponent) +
  labs(title="PCA 'small multiples' plot by date collected",
       subtitle = "all larvae included, NORMALIZED BY TUBE",
       caption=paste0("produced on ", Sys.time())) +
  theme_bw() +
  coord_flip()
```

### 5.2.2 Average brightness after normalizing by tube

When average brightness is considered, the tube / batch effects are very much mitigated by the normalization step. Going forward, I will work with normalized data. Of course, it would be better if every single brain could be collected on the same day and stained in the same tube, but we lacked the experimental throughput to do this.

```
average_signal <- rowMeans(dataOnlyNormed)

averageOnly <- cbind(studyDesign, average_signal) %>%
  filter(geno != "x") %>%
  filter(pheno != "MID") %>%
  filter(!(geno == "mut" & (collected == "210606" | collected == "210613")))

averageOnly$geno <- factor(averageOnly$geno, levels = c("WT", "het", "mut"))

#### Graph correlation between average brightness and phenotype ####

position <- position_jitter(0.1, 0)

# average_brightness_graph <- ggplot(averageOnly, aes(x = pheno, 
#                                                     y = average_signal, 
#                                                     color = collected)) +
#   geom_point(size = 2, position = position, alpha = 0.8) +
#  # scale_color_manual(values = c("red", "blue")) +
#   scale_y_continuous(limits = c(0,4), expand = c(0,0)) +
#   facet_wrap(~geno) +
#   ggtitle("Average CaSR-EGFP Signal Across All Brain Regions") +
#   labs(subtitle = "Weak stimulus") +
#   ylab("Average Normalized Signal Brightness \n(n = 254 brain regions, arbitrary units)") +
#   xlab("CaSR Genotype") +
#   labs(color = "Phenotype") +
#   theme(
#    # text=element_text(family="Arial"),
#     panel.background = element_blank(),
#     axis.line = element_line(color = "grey40"),
#     panel.grid.major.y = element_line(color = "grey90"),
#     strip.text.x = element_text(size = 12)
#   )
# 
# average_brightness_graph

average_brightness_bydate <- ggplot(averageOnly, aes(x = collected, 
                                                    y = average_signal, 
                                                    color = pheno)) +
  geom_point(size = 2, position = position, alpha = 0.8) +
  coord_flip() +
  scale_color_manual(values = c("red", "blue")) +
  scale_y_continuous(limits = c(0,4), expand = c(0,0)) +
 # facet_wrap(~geno) +
  ggtitle("Average NORMALIZED Signal") +
  labs(subtitle = "Weak stimulus") +
  ylab("Average Normalized Signal Brightness \n(n = 254 brain regions, arbitrary units)") +
  xlab("Date Collected") +
  labs(color = "Phenotype") +
  theme(
   # text=element_text(family="Arial"),
    panel.background = element_blank(),
    axis.line = element_line(color = "grey40"),
    panel.grid.major.y = element_line(color = "grey90"),
    strip.text.x = element_text(size = 12)
  )

average_brightness_bydate
```

## 5.3 Heat map on normalized data

Here, we visualize the signal intensity across a sample of brain regions to get a sense of patterns in the data. It seems that some brain regions are often bright, some are often dim, and most have a variety of signal intensity across different brains.

```
#filtering out pre-genotyped mutants used for rescue experiments from main dataset
normed_heatmap <- normed %>%
  filter(!(geno == "mut" & (collected == "210606" | collected == "210613"))) %>%
  filter(pheno != "MID") %>%
  filter(geno != "x")

studyDesign_heatmap <- studyDesign %>%
  filter(!(geno == "mut" & (collected == "210606" | collected == "210613"))) %>%
  filter(!(geno == "mut" & (collected == "210606" | collected == "210613"))) %>%
  filter(pheno != "MID") %>%
  filter(geno != "x")

normedDataOnly_Heatmap <- normed_heatmap[,8:length(normed_heatmap)]

regionNames <- colnames(normedDataOnly_Heatmap)

normedData_SelectRegions <- normedDataOnly_Heatmap %>%
  select(diencephalon_dorsal_thalamus,
         diencephalon_ventral_thalamus,
         diencephalon_habenula,
         ganglia_trigeminal_ganglion,
         ganglia_vagal_ganglia,
         mesencephalon_tecum_neuropil,
         mesencephalon_tegmentum,
         rhombencephalon_cerebellum,
         rhombencephalon_rhombomere_1,
         rhombencephalon_rhombomere_2,
         rhombencephalon_rhombomere_3,
         rhombencephalon_rhombomere_4,
         rhombencephalon_rhombomere_5,
         rhombencephalon_rhombomere_6,
         telencephalon_olfactory_bulb,
         telencephalon_pallium)

#heatmap function requires a matrix, so let's convert
heatmap_matrix <- as.matrix(normedData_SelectRegions)

#### Making a really nice heat map####
colores <- colorRamp2(c(0,3.5,8,15), c("black","green", "yellow","white")) #defining colors for the map itself

# Define colors for each levels of qualitative variables
# Define gradient color for continuous variable (mpg)
color_key = list(pheno = c("LLC" = "violetred4", "SLC" = "skyblue3"),
           geno = c("WT" = "blue", "het" = "green3", "mut" = "red"))
# Create the heatmap annotation
heatmap_annotation <- rowAnnotation(
  pheno = studyDesign_heatmap$pheno, geno = studyDesign_heatmap$geno,
  col = color_key
  )

annotated_all_larvae_heatmap <- Heatmap(heatmap_matrix,
        name = "CaSR-EGFP Signal", #title of legend
        column_title = "Brain Region", row_title = "Larva",
        col = colores,
        cluster_rows = FALSE,
        column_names_gp = gpar(fontsize = 8), # Text size for column names
        left_annotation = heatmap_annotation
)


clusteredHeatmap <- Heatmap(heatmap_matrix,
        name = "CaSR-EGFP Signal", #title of legend
        column_title = "Brain Region", row_title = "Larva",
        col = colores,
        column_names_gp = gpar(fontsize = 8) # Text size for column names
)

clusteredHeatmap
```

### 5.3.1 Heatmap with WT larvae only

To make the graph easier to read we also made a version with only WT larvae. The heatmap is sorted by the phenotype of the larva.

```
WT_heatmap <- normed_heatmap %>%
  filter(geno == "WT")

studyDesign_WT_heatmap <- WT_heatmap[1:7]

normedDataOnly_WT_heatmap <- WT_heatmap[,8:length(normed_heatmap)]

WT_heatmap_SelectRegions <- normedDataOnly_WT_heatmap %>%
  select(diencephalon_dorsal_thalamus,
         diencephalon_ventral_thalamus,
         diencephalon_habenula,
         ganglia_trigeminal_ganglion,
         ganglia_vagal_ganglia,
         mesencephalon_tecum_neuropil,
         mesencephalon_tegmentum,
         rhombencephalon_cerebellum,
         rhombencephalon_rhombomere_1,
         rhombencephalon_rhombomere_2,
         rhombencephalon_rhombomere_3,
         rhombencephalon_rhombomere_4,
         rhombencephalon_rhombomere_5,
         rhombencephalon_rhombomere_6,
         telencephalon_olfactory_bulb,
         telencephalon_pallium)

WT_heatmap_SelectRegions_matrix <- as.matrix(WT_heatmap_SelectRegions)

color_key_WT <- list(pheno = c("LLC" = "#AA6364", "SLC" = "#3E6BA1"))

heatmap_annotation_WTs <- rowAnnotation(
  pheno = studyDesign_WT_heatmap$pheno,
  col = color_key_WT
)

Heatmap(WT_heatmap_SelectRegions_matrix,
        name = "CaSR-EGFP Signal", #title of legend
        column_title = "Brain Region", row_title = "Larva",
        col = colores,
        cluster_rows = FALSE,
        column_names_gp = gpar(fontsize = 6), # Text size for column names
        left_annotation = heatmap_annotation_WTs
)
```

## 5.4 Correlational analysis on normalized data

If signal in two brain regions perfectly correlates, then their individual influences on a larva’s phenotype will be impossible to disentangle. Here we determine how much correlation is present between brain regions in our dataset. We see that certain pairs of brain regions are highly correlated with one another, but that other pairs of brain regions are not as highly correlated. This allows the possibility that an individual brain region or region(s) will correlate more strongly with phenotype than the others. However, it also raises the possibility that any highly correlated (with phenotype) region will also have other highly correlated (with the highly correlated region) regions. This emphasizes the need for independent experimental validation of the results of multivariate analysis (e.g. by testing rescue and/or sufficiency to produce gain-of-function phenotype with more specific Gal4s targeting the region of interest).

```
### Making correlation plot with only selected regions 1.23.22
normed_corr_select <- normed_heatmap %>%
  select(diencephalon_dorsal_thalamus,
         diencephalon_ventral_thalamus,
         diencephalon_habenula,
         ganglia_trigeminal_ganglion,
         ganglia_vagal_ganglia,
         ganglia_anterior_lateral_line_ganglion,
         ganglia_posterior_lateral_line_ganglia,
         mesencephalon_tecum_neuropil,
         mesencephalon_tegmentum,
         rhombencephalon_cerebellum,
         rhombencephalon_locus_coreuleus,
         rhombencephalon_mauthner,
         rhombencephalon_rhombomere_1,
         rhombencephalon_rhombomere_2,
         rhombencephalon_rhombomere_3,
         rhombencephalon_rhombomere_4,
         rhombencephalon_rhombomere_5,
         rhombencephalon_rhombomere_6,
         rhombencephalon_raphe_inferior,
         rhombencephalon_raphe_superior,
         telencephalon_olfactory_bulb,
         telencephalon_pallium)

corr_select <- cor(normed_corr_select)

#corr_select_df <- as.data.frame(corr_select)

#write_csv(corr_select_df, file = "PairwisePearsonCorrs_RawData.csv")

#A different version of the correlation plot with no labels, color-coded only

# plain_corr_plot_select <- corrplot(corr_select,
#                                    method = 'color',
#                                    tl.pos = "n",
#                                    order = 'hclust',
#                                    hclust.method = "ward.D2")

# Final version of the corrplot uses ellipses to demonstrate strength 

corrplot(corr_select, method = 'ellipse', order = 'AOE', type = 'lower', tl.col = 'black')
```

# 6 LASSO Regression

We have seen that average signal is not very informative regarding whether a CaSR WT or het larva will be biased towards LLCs or SLCs. However, there may be individual brain regions that are predictive of CaSR WT or het behavior. To identify these brain regions, I perform a multivariate analysis. Because I have more brain regions (n=251) than WT or het larvae, I need to manage overfitting. To do this, I have chosen to perform a form of logistic regression that applies a penalty to assignment of coefficients. Specifically, LASSO logistic regression allows coefficients to go to 0, resulting in a model that incorporates few brain regions and is easily interpretable.

First, we perform cross-validation to determine what a good value for lambda (the weight of the penalty applied to coefficients) is. We run lasso logistic regression on the entire dataset to calculate coefficients. Then we bootstrap to create many models and assess their accuracy, just like we did for the univariate logistic regression on average brightness. This can help us assess the uncertainty associated with the coefficients generated from the whole dataset.

## 6.1 Step 1: Finding lambda

```
#select only WTs for building lasso regression model
WTs <- normed %>%
  filter(geno == "WT") %>%
  filter(pheno != "MID") %>%
  mutate(pheno = recode(pheno, "LLC" = 0, "SLC" = 1))   #recode so lasso function doesn't throw a fit

WTDesign <- WTs[, 1:7]

WTNumeric <- WTs%>%
  select(-unique_id, -fish_num, -pair,-geno, -imaged, -collected)

#### turn into a matrix using model.matrix ####

x = model.matrix(pheno~.,WTNumeric)[,-1]
y = WTNumeric$pheno

#### for loop to find the best lambda ####

seed = 0
biglist <- list()

#### for loop to find the best lambda ####

seed = 0
trial = 0
biglist <- list()

for (trial in 0:300) {

  # randomly subset the data, taking 80% (essentially doing 5-fold cross-validation)  
  set.seed(seed)
  train = sample(c(TRUE,TRUE,TRUE,TRUE,FALSE), nrow(WTNumeric), rep = TRUE) 
  test = (!train)
  test
  train
  
  # perform 4-fold cross-validation to identify the best value for lambda

  y[train]
  sum(y[train] == 0)
  if (sum(y[train] == 0) < 8) {       #the cross-validation can't handle having 0 in either category. Making sure you have at least 8 in each category before cross-validating makes that much less likely
    print("Skipping this seed - too few LLCs")
    seed = seed + 1
    
  } else if (sum(y[train] == 1) < 8) {
    print("Skipping this seed - too few SLCs")  #ditto
    seed = seed + 1
    
  } else {
  cv.out = cv.glmnet(x[train,], y[train], alpha = 1, family = "binomial", nfolds = 4)  # logistic regression with lasso regularization
  bestlam = cv.out$lambda.min
  
  results <- c(seed, bestlam)
  biglist <- c(biglist, results)
  
  seed = seed + 1
  trial = trial + 1  #only advance progression through the loop if you found a seed that worked
  }
  
}
```

```
## [1] "Skipping this seed - too few LLCs"
## [1] "Skipping this seed - too few LLCs"
## [1] "Skipping this seed - too few LLCs"
## [1] "Skipping this seed - too few LLCs"
## [1] "Skipping this seed - too few LLCs"
```

```
lambda_bootstrap <- matrix(unlist(biglist), ncol = 2, byrow = TRUE)
colnames(lambda_bootstrap) <- c("Seed", "BestLam")

lambda_bootstrap <- as_tibble(lambda_bootstrap)
avg_bestlam <- mean(lambda_bootstrap$BestLam)
```

## 6.2 Step 2: LASSO regression on WT larvae

For lambda, we will be using the value 0.1335029, which we generated by cross-validation in the previous step.

```
#### do the lasso regression and get coefficients on the ENTIRE WT DATASET ####
grid = 10^seq(10, -2, length = 100)
output = glmnet(x, y, alpha = 1, lambda = grid)
lasso_coeff = predict(output, type = "coefficients", s = avg_bestlam)
nonzero_coeffs <- lasso_coeff[lasso_coeff!=0]

lasso_coeff <- as_tibble(as.matrix(lasso_coeff))

#make presentable

coeff_length <- length(lasso_coeff)

lasso_coeff_with_regions <- lasso_coeff[2:(length(regionNames)+1),1]
lasso_coeff_with_regions$Region <- regionNames
colnames(lasso_coeff_with_regions) <- c("Coefficient", "Region")

nonzero_regions <- lasso_coeff_with_regions %>%
  filter(Coefficient != 0) %>%
  relocate(Region)

nonzero_regions
```

```
## # A tibble: 1 x 2
##   Region                                     Coefficient
##   <chr>                                            <dbl>
## 1 rhombencephalon_qrfp_neuron_cluster_sparse      0.0416
```

```
regions <- nonzero_regions[1]
coeff_assigned <- nonzero_regions[2]

gt_table <- gt(nonzero_regions) 
gt_table
```

| Region | Coefficient |
| --- | --- |
| rhombencephalon\_qrfp\_neuron\_cluster\_sparse | 0.04157285 |

## 6.3 Lasso regression: initial findings implicate the Rhombencephalon QRFP Cluster - Sparse

Lasso regression on the full dataset identified that CaSR expression levels in rhombencephalon\_qrfp\_neuron\_cluster\_sparse can potentially predict whether a WT larva performs LLCs or SLCs, with coefficient value of 0.0415729.

In this analysis, we *exclude* the following brains:

- those that were not successfully genotyped (genotype coded as “x”)
- those that did not display a clear bias towards either the SLC or LLC phenotype (phenotype coded as “MID”)
- those belonging to CaSR mutants that were pre-genotyped and used for RESCUE, rather than gain-of-function, experiments

```
rhomb_QRFP_signal <- (normed) %>%
  select(pheno,geno, collected, rhombencephalon_qrfp_neuron_cluster_sparse) %>%
  filter(geno != "x") %>%
  filter(!((geno == "mut")  & (collected == "210606" | collected == "210613"))) %>% #these mutants were responding to high-intensity stimuli and should NOT be included in the low-intensity stimulus analysis
  filter(pheno != "MID")

signal_strongStim <- (normed) %>%
  select(pheno,geno, collected, rhombencephalon_qrfp_neuron_cluster_sparse, rhombencephalon_locus_coreuleus) %>%
  filter(geno != "x") %>%
  filter((geno == "mut")  & (collected == "210606" | collected == "210613")) %>% 
  filter(pheno != "MID")

write_csv(signal_strongStim, "StrongStimSelectedSignal.csv")

rhomb_QRFP_signal_means <- rhomb_QRFP_signal %>%
  group_by(geno, pheno) %>%
  summarise(mean(rhombencephalon_qrfp_neuron_cluster_sparse)) 

rhomb_QRFP_signal_sd <- rhomb_QRFP_signal %>%
  group_by(geno, pheno) %>%
  summarise(sd(rhombencephalon_qrfp_neuron_cluster_sparse)) 

rhomb_QRFP_signal_n <- rhomb_QRFP_signal %>%
  count(geno, pheno) 

rhomb_QRFP_signal_min <- rhomb_QRFP_signal %>%
  group_by(geno, pheno) %>%
  summarise(min(rhombencephalon_qrfp_neuron_cluster_sparse)) 

# WRONG -- DO NOT USE #write_csv(rhomb_QRFP_signal, "DCR6_Signal_CorrectNorm_220224.csv")

#use me
#write.csv(rhomb_QRFP_signal, "DCR6_Signal_CorrectNorm_220228_writeDOTcsv.csv")

rhomb_QRFP_signal$geno <- factor(rhomb_QRFP_signal$geno, levels = c("mut", "het", "WT"))
position = position_jitter(0.1,0)

Rhomb_QRFP_brightness_graph <- ggplot(rhomb_QRFP_signal, aes(x = pheno, 
                                                             y = rhombencephalon_qrfp_neuron_cluster_sparse,
                                                             color = pheno)) +
  geom_point(size = 2, position = position, alpha = 0.8) +
  scale_color_manual(values = c("#AA6364","#3e6BA1")) +
  scale_y_continuous(limits = c(0,16), expand = c(0,0)) +
  facet_wrap(~geno) +
  ggtitle("Brightness of Rhombencephalon QRFP Cluster - Sparse") +
  ylab("Average Normalized Signal Brightness \nRhombencephalon QRFP Cluster - Sparse (arbitrary units)") +
  xlab("CaSR Genotype") +
  labs(color = "Phenotype") +
  theme(
    text=element_text(family="Arial"),
    panel.background = element_blank(),
    axis.line = element_line(color = "grey40"),
    panel.grid.major.y = element_line(color = "grey90"),
    strip.text.x = element_text(size = 8)
  )

Rhomb_QRFP_brightness_graph
```

### 6.3.1 Control region: Locus Coeruleus

We picked the Locus Coeruleus as a control region of comparable size and in the same general region (hindbrain) as the Rhombencephalon QRFP Cluster - Sparse. From visual inspection, it is clear that there is not as strong a relationship between phenotype and signal in the LC as there was in the DCR6.

```
#Note: "locus coeruleus" is misspelled in the whole brain atlas. You must spell it wrong to get the correct column

LC_signal <- (normed) %>%
  select(pheno,geno, collected, rhombencephalon_locus_coreuleus) %>%
  filter(geno != "x") %>%
  filter(!((geno == "mut")  & (collected == "210606" | collected == "210613"))) %>% #these mutants were responding to high-intensity stimuli and should NOT be included in the low-intensity stimulus analysis
  filter(pheno != "MID")

write.csv(rhomb_QRFP_signal, "LC_Signal_CorrectNorm_220228_writeDOTcsv.csv")

LC_signal$geno <- factor(LC_signal$geno, levels = c("mut", "het", "WT"))
position = position_jitter(0.1,0)

LC_brightness_graph <- ggplot(LC_signal, aes(x = pheno, 
                                                             y = rhombencephalon_locus_coreuleus,
                                                             color = pheno)) +
  geom_point(size = 2, position = position, alpha = 0.8) +
  scale_color_manual(values = c("#AA6364","#3e6BA1")) +
  scale_y_continuous(limits = c(0,4), expand = c(0,0)) +
  facet_wrap(~geno) +
  ggtitle("Brightness of the Locus Coeruleus") +
  ylab("Average Normalized Signal Brightness \nLocus Coeruleus (arbitrary units)") +
  xlab("CaSR Genotype") +
  labs(color = "Phenotype") +
  theme(
    text=element_text(family="Arial"),
    panel.background = element_blank(),
    axis.line = element_line(color = "grey40"),
    panel.grid.major.y = element_line(color = "grey90"),
    strip.text.x = element_text(size = 8)
  )

LC_brightness_graph
```

### 6.3.2 Two-way ANOVA to confirm significant association between signal in brain region and phenotype

The LASSO regression on WT larvae has identified a candidate brain region, but to confirm that signal in that brain region is associated with phenotype across the whole dataset, we perform a two-way ANOVA analysis with independent variables of phenotype and genotype and dependent variable of CaSR-EGFP signal in the Rhombencephalon QRFP Cluster. This analysis indicates a highly significant association between signal in the Rhombencephalon QRFP Cluster and phenotype (main effect of phenotype).

```
rhomb_QRFP_signal <- normed %>%
  filter(geno != "x") %>%
  filter(pheno != "MID") %>%
  filter(!((geno == "mut")  & (collected == "210606" | collected == "210613"))) %>%
  select(pheno,geno,rhombencephalon_qrfp_neuron_cluster_sparse) 

rhomb_QRFP_counts <- rhomb_QRFP_signal %>%
  count(geno,pheno)

# Perform two-way ANOVA
rhomb_qrfp_2way <- aov(rhombencephalon_qrfp_neuron_cluster_sparse ~ pheno * geno, data=rhomb_QRFP_signal) 
summary(rhomb_qrfp_2way)
```

```
##              Df Sum Sq Mean Sq F value  Pr(>F)   
## pheno         1   41.4   41.36   8.516 0.00413 **
## geno          2    8.0    3.98   0.820 0.44250   
## pheno:geno    2   42.7   21.34   4.393 0.01419 * 
## Residuals   134  650.8    4.86                   
## ---
## Signif. codes:  0 '***' 0.001 '**' 0.01 '*' 0.05 '.' 0.1 ' ' 1
```

### 6.3.3 Two-way ANOVA on control region

The effect of phenotype, genotype, and phenotype x genotype interaction are all not significant in the LC.

```
# Perform two-way ANOVA
LC_2way <- aov(rhombencephalon_locus_coreuleus ~ pheno * geno, data= LC_signal) 
summary(LC_2way)
```

```
##              Df Sum Sq Mean Sq F value Pr(>F)  
## pheno         1   1.29  1.2912   3.493 0.0638 .
## geno          2   0.26  0.1317   0.356 0.7010  
## pheno:geno    2   0.15  0.0730   0.197 0.8212  
## Residuals   134  49.54  0.3697                 
## ---
## Signif. codes:  0 '***' 0.001 '**' 0.01 '*' 0.05 '.' 0.1 ' ' 1
```

## 6.4 Bootstrap analysis to evaluate predictive ability of LASSO-identified region vs. average signal

LASSO regression has identified the DCR6 (a.k.a. Rhombencephlan QRFP Cluster - Sparse) as a region where CaSR-EGFP signal can predict a WT or mutant larva’s phenotype. However, correlation analysis indicates that all of the brain regions have greater or lesser degree of correlation with one another. It is possible that CaSR across the whole brain exerts some gross effect on CSF calcium levels, thereby altering behavior, and that the signal in the DCR6 is merely a particularly good proxy for CaSR signal in the whole brain. In this section, we compare the ability of average CaSR-EGFP signal in the brain vs. signal in the DCR6 to predict larval phenotype.

### 6.4.1 Correlation between average signal and signal in the DCR6

To investigate this possibility, first we examined the correlation between average CaSR-EGFP signal across the whole brain and CaSR-EGFP signal particularly in the DCR6. In WT, heterozygous, and mutant larvae, this correlation was clear, but not particularly strong compared to other correlations we observed between individual brain regions.

```
average_and_QRFP <- averageOnly 

QRFPOnly <- normed %>%
  select(unique_id, fish_num, pair, geno, pheno, collected, imaged, rhombencephalon_qrfp_neuron_cluster_sparse)

QRFPOnly <- QRFPOnly %>%
  filter(geno != "x") %>%
  filter(pheno != "MID") %>%
  filter(!(geno == "mut" & (collected == "210606" | collected == "210613")))

average_and_QRFP$rhombQRFP <- QRFPOnly$rhombencephalon_qrfp_neuron_cluster_sparse

average_and_QRFP <- average_and_QRFP %>%
  mutate(geno = as.factor(geno)) %>%
  arrange(geno)

average_and_QRFP_recoded <- average_and_QRFP %>%
  mutate(geno = recode(geno, WT = "+/+", het = "+/-", mut = "-/-"))

corr_title <- expression(paste(italic("CaSR"), " Genotype"))

corr_by_geno <- ggplot(average_and_QRFP_recoded, aes(x = average_signal, y = rhombQRFP, color = pheno)) +
  facet_wrap(~geno) +
  ggtitle(corr_title) +
  geom_point() +
  scale_color_manual(values = c("#AA6364", "#3E6BA1"), labels = c("Reor. Bias", "Esc. Bias")) +
  scale_y_continuous("CaSR-EGFP Signal in DCR6", 
                    # breaks = c(0,.25,.50,.75,1),
                     limits = c(0,15),
                     expand = c(0,0.1)) +
  scale_x_continuous("Average CaSR-EGFP Signal Across All Brain Regions") +
  labs(color = "Phenotype") +
    theme(
        plot.title = element_text(size = 12, face = "bold", hjust = 0.5),
        axis.text.x = element_text(color = "black", size = 8, angle = 45, hjust = 1),
        axis.line = element_line(color = "black"),
       # axis.text.x = element_blank(),
        axis.text.y = element_text(color = "black", size = 8),
        panel.grid.major.y = element_blank(),
        panel.grid.major.x = element_blank(),
        panel.background = element_rect(fill = NA),
        #legend.position = "none",
        axis.title.y = element_text(size=10, face = "bold"),
        axis.title.x = element_text(size=10, face = "bold"),
        strip.text = element_text(size = 10))

corr_by_geno
```

```
#ggsave("CaSRMut_Avg_DCR6_Corr.png",
  #     device = png,
  #     units = "px",
  #     width = 2000,
  #     height = 1000,
  #     dpi = 300)
```

### 6.4.2 Measuring predictive ability of average signal in the brain

#### 6.4.2.1 Bootstrapping WT Logistic Regression

First, I filter so that I only have WT larvae that did LLCs or SLCs. Then, I take a random 50% of those larvae (the training set) and I use them to perform logistic regression on average signal vs. phenotype. I then check how well that logistic regression equation is able to predict the phenotype of the other 50% of my larvae (the testing set). That gives me a sense of how accurate the model is, but it might be too specific to the particular training and testing sets of larvae that I randomly selected. By repeating the process 100 times, each with a different random subset of my larvae used for testing and training, I’m able to get a more general sense of how accurate my model is.

```
#### Filter for just hets with LLC or SLC phenotype (very low n with "mid" pheno)

WT_average <- averageOnly %>%
  filter(geno == "WT") %>%
  filter(pheno != "MID")

WT_average$pheno <- as.factor(WT_average$pheno)

#bootstrapping logistic regression
seed = 0
biglist <- list()
sample_size = ceiling(0.5*nrow(WT_average))

# within for loop
for (seed in 1:100) {
  # randomly split data 
  chosen = sample(seq_len(nrow(WT_average)), size = sample_size)
  glm.training =WT_average[chosen,]
  glm.testing =WT_average[-chosen,]
  
  #fit model
  glm.fit <- glm(pheno ~ average_signal, data = glm.training, family = binomial)

  #make predictions
  glm.probs <- predict(glm.fit,
                       newdata = glm.testing,
                       type = "response")

  # contrasts(glm.testing$pheno)# tells me which value has been assigned to which phenotype

  Predicted <- ifelse(glm.probs > 0.5, "SLC", "LLC")

  Actual <- glm.testing$pheno

  ConfusionMatrix <- table(Predicted, Actual)
  ConfusionMatrix

  accuracy <- sum(diag(ConfusionMatrix)) / sum(ConfusionMatrix)

  intercept <- glm.fit$coefficients[1]
  bright_coef <- glm.fit$coefficients[2]

  results <- c(seed, intercept, bright_coef, accuracy)

  biglist <- c(biglist, results)
  seed = seed + 1
}

#out of for loop

bootstrap_result_WT <- matrix(unlist(biglist), ncol = 4, byrow = TRUE)
colnames(bootstrap_result_WT) <- c("Seed", "Intercept", "Brightness_Coeff", "Accuracy")

bootstrap_result_WT <- as_tibble(bootstrap_result_WT)

avg_accuracy_WT <- mean(bootstrap_result_WT$Accuracy)
#avg_accuracy_WT
sd_accuracy_WT <- sd(bootstrap_result_WT$Accuracy) 
#sd_accuracy_WT
```

#### 6.4.2.2 Interpreting WT Logistic Regression Results

The average testing accuracy of a logistic regression model constructed using only data from WT larvae is 0.4548. Thus, this model does not accurately predict whether WT larvae will perform SLCs or LLCs.

#### 6.4.2.3 Bootstrapping Heterozygote Logistic Regression

Next, we see if we can do any better on the heterozygotes.

```
het_average <- averageOnly %>%
  filter(geno == "het") %>%
  filter(pheno != "MID")

het_average$pheno <- as.factor(het_average$pheno)

#bootstrapping logistic regression
seed = 0
biglist <- list()
sample_size = ceiling(0.5*nrow(het_average))

# within for loop
for (seed in 1:100) {
  set.seed(seed)
  
  # randomly split data 

  chosen = sample(seq_len(nrow(het_average)),size = sample_size)
  glm.training = het_average[chosen,]
  glm.testing = het_average[-chosen,]
  
  #fit model
  glm.fit <- glm(pheno ~ average_signal, data = glm.training, family = binomial)

  glm.probs <- predict(glm.fit,
                       newdata = glm.testing,
                       type = "response")

  # contrasts(glm.testing$pheno)# tells me which value has been assigned to which phenotype

  Predicted <- ifelse(glm.probs > 0.5, "SLC", "LLC")

  Actual <- glm.testing$pheno

  ConfusionMatrix <- table(Predicted, Actual)
  ConfusionMatrix

  accuracy <- sum(diag(ConfusionMatrix)) / sum(ConfusionMatrix)

  intercept <- glm.fit$coefficients[1]
  bright_coef <- glm.fit$coefficients[2]

  results <- c(seed, intercept, bright_coef, accuracy)

  biglist <- c(biglist, results)
  seed = seed + 1
}

#out of for loop

bootstrap_result_het <- matrix(unlist(biglist), ncol = 4, byrow = TRUE)
colnames(bootstrap_result_het) <- c("Seed", "Intercept", "Brightness_Coeff", "Accuracy")

bootstrap_result_het <- as_tibble(bootstrap_result_het)

avg_accuracy_het <- mean(bootstrap_result_het$Accuracy)
#avg_accuracy_het
sd_accuracy_het <- sd(bootstrap_result_het$Accuracy)
#sd_accuracy_het
```

#### 6.4.2.4 Interpreting Heterozygote Logistic Regression Results

The average testing accuracy of a logistic regression model constructed using only average signal data from CaSR heterozygous larvae is 0.4805128. Thus, this model is slightly better than chance at predicting whether heterozygous larvae will perform SLCs or LLCs.

#### 6.4.2.5 Bootstrapping Mutant Logistic Regression

Let’s try one more time with the mutants!

```
mut_average <- averageOnly %>%
  filter(geno == "mut") %>%
  filter(pheno != "MID")

mut_average$pheno <- as.factor(mut_average$pheno)

#bootstrapping logistic regression
seed = 0
biglist <- list()
sample_size = ceiling(0.5*nrow(mut_average))

# within for loop
for (seed in 1:100) {
  set.seed(seed)
  
  # randomly split data 

  chosen = sample(seq_len(nrow(mut_average)),size = sample_size)
  glm.training = mut_average[chosen,]
  glm.testing = mut_average[-chosen,]
  
  #fit model
  glm.fit <- glm(pheno ~ average_signal, data = glm.training, family = binomial)

  glm.probs <- predict(glm.fit,
                       newdata = glm.testing,
                       type = "response")

  # contrasts(glm.testing$pheno)# tells me which value has been assigned to which phenotype

  Predicted <- ifelse(glm.probs > 0.5, "SLC", "LLC")

  Actual <- glm.testing$pheno

  ConfusionMatrix <- table(Predicted, Actual)
  ConfusionMatrix

  accuracy <- sum(diag(ConfusionMatrix)) / sum(ConfusionMatrix)

  intercept <- glm.fit$coefficients[1]
  bright_coef <- glm.fit$coefficients[2]

  results <- c(seed, intercept, bright_coef, accuracy)

  biglist <- c(biglist, results)
  seed = seed + 1
}

#out of for loop

bootstrap_result_mut <- matrix(unlist(biglist), ncol = 4, byrow = TRUE)
colnames(bootstrap_result_mut) <- c("Seed", "Intercept", "Brightness_Coeff", "Accuracy")

bootstrap_result_mut <- as_tibble(bootstrap_result_mut)

avg_accuracy_mut <- mean(bootstrap_result_mut$Accuracy)
#avg_accuracy_mut
sd_accuracy_mut <- sd(bootstrap_result_mut$Accuracy)
#sd_accuracy_mut
```

#### 6.4.2.6 Interpreting Mutant Logistic Regression Results

The average testing accuracy of a logistic regression model constructed using only average signal data from CaSR mutant larvae is 0.594. This suggests that the average CaSR-EGFP signal in a mutant brain is a fairly good predictor of whether or not that mutant’s behavioral phenotype will be shifted.

```
#Now I put all the results from the logistic regressions into a single table
genotypes_longhand <- c("+/+","+/-","-/-")
logistic_accuracy <- c(avg_accuracy_WT, avg_accuracy_het, avg_accuracy_mut)
logistic_accuracy_sd <- c(sd_accuracy_WT, sd_accuracy_het, sd_accuracy_mut)
model_avg <- c("Whole-brain average", "Whole-brain average", "Whole-brain average")
logistic_accuracy_tibble <- tibble(genotypes_longhand, model_avg, logistic_accuracy,logistic_accuracy_sd)
colnames(logistic_accuracy_tibble) = (c("Genotype","Model","Accuracy","StDev"))
gt_table_avgSig <- gt(logistic_accuracy_tibble) 
gt_table_avgSig
```

| Genotype | Model | Accuracy | StDev |
| --- | --- | --- | --- |
| +/+ | Whole-brain average | 0.4548000 | 0.18322851 |
| +/- | Whole-brain average | 0.4805128 | 0.07597796 |
| -/- | Whole-brain average | 0.5940000 | 0.19375086 |

### 6.4.3 Measuring predictive ability of Rhombencephalon QRFP Cluster / DCR6

The LASSO regression has identified that signal in the Rhombencephalon QRFP Cluster is the best predictor of phenotype of any individual brain region, at least in WT animals. We now wish to compare the predictive ability of the DCR6 to the predictive ability of the average signal in the brain.

Let’s start with WT larvae…

```
QRFPOnly <- normed %>%
  select(unique_id, fish_num, pair, geno, pheno, collected, imaged, rhombencephalon_qrfp_neuron_cluster_sparse)

WT_QRFP <- QRFPOnly %>%
  filter(geno == "WT") %>%
  filter(pheno != "MID")

WT_QRFP$pheno <- as.factor(WT_QRFP$pheno)

#bootstrapping logistic regression
seed = 0
biglist <- list()
sample_size = ceiling(0.5*nrow(WT_QRFP))

# within for loop
for (seed in 1:100) {
  set.seed(seed)
  
  # randomly split data 

  chosen = sample(seq_len(nrow(WT_QRFP)),size = sample_size)
  glm.training = WT_QRFP[chosen,]
  glm.testing = WT_QRFP[-chosen,]
  
  #fit model
  glm.fit <- glm(pheno ~ rhombencephalon_qrfp_neuron_cluster_sparse, data = glm.training, family = binomial)

  glm.probs <- predict(glm.fit,
                       newdata = glm.testing,
                       type = "response")

  # contrasts(glm.testing$pheno)# tells me which value has been assigned to which phenotype

  Predicted <- ifelse(glm.probs > 0.5, "SLC", "LLC")

  Actual <- glm.testing$pheno

  ConfusionMatrix <- table(Predicted, Actual)
  ConfusionMatrix

  accuracy <- sum(diag(ConfusionMatrix)) / sum(ConfusionMatrix)

  intercept <- glm.fit$coefficients[1]
  bright_coef <- glm.fit$coefficients[2]

  results <- c(seed, intercept, bright_coef, accuracy)

  biglist <- c(biglist, results)
  seed = seed + 1
}

#out of for loop

bootstrap_result_QRFP_WT <- matrix(unlist(biglist), ncol = 4, byrow = TRUE)
colnames(bootstrap_result_QRFP_WT) <- c("Seed", "Intercept", "Brightness_Coeff", "Accuracy")

bootstrap_result_QRFP_WT <- as_tibble(bootstrap_result_QRFP_WT)

avg_accuracy_QRFP_WT <- mean(bootstrap_result_QRFP_WT$Accuracy)
#avg_accuracy_QRFP_WT
sd_accuracy_QRFP_WT <- sd(bootstrap_result_QRFP_WT$Accuracy)
#sd_accuracy_QRFP_WT
```

#### 6.4.3.1 QRFP Logistic Analysis, Hets

Now let’s move to hets…

```
het_QRFP <- QRFPOnly %>%
  filter(geno == "het") %>%
  filter(pheno != "MID")

het_QRFP$pheno <- as.factor(het_QRFP$pheno)

#bootstrapping logistic regression
seed = 0
biglist <- list()
sample_size = ceiling(0.5*nrow(het_QRFP))

# within for loop
for (seed in 1:100) {
  set.seed(seed)
  
  # randomly split data 

  chosen = sample(seq_len(nrow(het_QRFP)),size = sample_size)
  glm.training = het_QRFP[chosen,]
  glm.testing = het_QRFP[-chosen,]
  
  #fit model
  glm.fit <- glm(pheno ~ rhombencephalon_qrfp_neuron_cluster_sparse, data = glm.training, family = binomial)

  glm.probs <- predict(glm.fit,
                       newdata = glm.testing,
                       type = "response")

  # contrasts(glm.testing$pheno)# tells me which value has been assigned to which phenotype

  Predicted <- ifelse(glm.probs > 0.5, "SLC", "LLC")

  Actual <- glm.testing$pheno

  ConfusionMatrix <- table(Predicted, Actual)
  ConfusionMatrix

  accuracy <- sum(diag(ConfusionMatrix)) / sum(ConfusionMatrix)

  intercept <- glm.fit$coefficients[1]
  bright_coef <- glm.fit$coefficients[2]

  results <- c(seed, intercept, bright_coef, accuracy)

  biglist <- c(biglist, results)
  seed = seed + 1
}

#out of for loop

bootstrap_result_QRFP_het <- matrix(unlist(biglist), ncol = 4, byrow = TRUE)
colnames(bootstrap_result_QRFP_het) <- c("Seed", "Intercept", "Brightness_Coeff", "Accuracy")

bootstrap_result_QRFP_het <- as_tibble(bootstrap_result_QRFP_het)

avg_accuracy_QRFP_het <- mean(bootstrap_result_QRFP_het$Accuracy)
#avg_accuracy_QRFP_het
sd_accuracy_QRFP_het <- sd(bootstrap_result_QRFP_het$Accuracy)
#sd_accuracy_QRFP_het
```

#### 6.4.3.2 QRFP Logistic Analysis, muts

And finally, mutants…

```
QRFPOnly <- normed %>%
  select(unique_id, fish_num, pair, geno, pheno, collected, imaged, rhombencephalon_qrfp_neuron_cluster_sparse)

mut_QRFP <- QRFPOnly %>%
  filter(geno == "mut") %>%
  filter(pheno != "MID")

mut_QRFP$pheno <- as.factor(mut_QRFP$pheno)

#bootstrapping logistic regression
seed = 0
biglist <- list()
sample_size = ceiling(0.5*nrow(mut_QRFP))

# within for loop
for (seed in 1:100) {
  set.seed(seed)
  
  # randomly split data 

  chosen = sample(seq_len(nrow(mut_QRFP)),size = sample_size)
  glm.training = mut_QRFP[chosen,]
  glm.testing = mut_QRFP[-chosen,]
  
  #fit model
  glm.fit <- glm(pheno ~ rhombencephalon_qrfp_neuron_cluster_sparse, data = glm.training, family = binomial)

  glm.probs <- predict(glm.fit,
                       newdata = glm.testing,
                       type = "response")

  # contrasts(glm.testing$pheno)# tells me which value has been assigned to which phenotype

  Predicted <- ifelse(glm.probs > 0.5, "SLC", "LLC")

  Actual <- glm.testing$pheno

  ConfusionMatrix <- table(Predicted, Actual)
  ConfusionMatrix

  accuracy <- sum(diag(ConfusionMatrix)) / sum(ConfusionMatrix)

  intercept <- glm.fit$coefficients[1]
  bright_coef <- glm.fit$coefficients[2]

  results <- c(seed, intercept, bright_coef, accuracy)

  biglist <- c(biglist, results)
  seed = seed + 1
}

#out of for loop

bootstrap_result_QRFP_mut <- matrix(unlist(biglist), ncol = 4, byrow = TRUE)
colnames(bootstrap_result_QRFP_mut) <- c("Seed", "Intercept", "Brightness_Coeff", "Accuracy")

bootstrap_result_QRFP_mut <- as_tibble(bootstrap_result_QRFP_mut)

avg_accuracy_QRFP_mut <- mean(bootstrap_result_QRFP_mut$Accuracy)
#avg_accuracy_QRFP_mut
sd_accuracy_QRFP_mut <- sd(bootstrap_result_QRFP_mut$Accuracy)
#sd_accuracy_QRFP_mut

logistic_QRFP_accuracy <- c(avg_accuracy_QRFP_WT, avg_accuracy_QRFP_het, avg_accuracy_QRFP_mut)
logistic_QRFP_sd <- c(sd_accuracy_QRFP_WT, sd_accuracy_QRFP_het, sd_accuracy_QRFP_mut)
model_QRFP <- c("DCR6", "DCR6", "DCR6")
logistic_QRFP_tibble <- tibble(genotypes_longhand, model_QRFP, logistic_QRFP_accuracy,logistic_QRFP_sd)
colnames(logistic_QRFP_tibble) = (c("Genotype","Model","Accuracy","StDev"))
gt_table_QRFP <- gt(logistic_QRFP_tibble) 
gt_table_QRFP
```

| Genotype | Model | Accuracy | StDev |
| --- | --- | --- | --- |
| +/+ | DCR6 | 0.6888000 | 0.14501327 |
| +/- | DCR6 | 0.4961538 | 0.07639245 |
| -/- | DCR6 | 0.7445455 | 0.10312344 |

### 6.4.4 Multiple logistic regression with average signal + DCR6

As we showed above, average CaSR-EGFP signal and DCR6 CaSR-EGFP signal are correlated with one another, but not very strongly. Therefore, it is unlikely that these two variables are totally redundant. A multiple regression using both variables might therefore provide an improved prediction over either single regression (with average signal or DCR6 signal alone). We will therefore add one more model to our comparison: a model that incorporates both average signal and DCR6 signal in a multiple logistic regression.

#### 6.4.4.1 WT larvae

Bootstrapping on WT larvae…

```
WT_average_and_QRFP <- average_and_QRFP %>%
  filter(geno == "WT") 

WT_average_and_QRFP$pheno <- as.factor(WT_average_and_QRFP$pheno)

#bootstrapping logistic regression
seed = 0
biglist <- list()
sample_size = ceiling(0.5*nrow(WT_average_and_QRFP))

# within for loop
for (seed in 1:100) {
  set.seed(seed)
  
  # randomly split data 

  chosen = sample(seq_len(nrow(WT_average_and_QRFP)),size = sample_size)
  glm.training = WT_average_and_QRFP[chosen,]
  glm.testing = WT_average_and_QRFP[-chosen,]
  
  #fit model
  #multiple regression this time!
  glm.fit <- glm(pheno ~ average_signal + rhombQRFP, data = glm.training, family = binomial) 
  
  glm.probs <- predict(glm.fit,
                       newdata = glm.testing,
                       type = "response")
  
  # contrasts(glm.testing$pheno)# tells me which value has been assigned to which phenotype
  
  Predicted <- ifelse(glm.probs > 0.5, "SLC", "LLC")
  
  Actual <- glm.testing$pheno
  
  ConfusionMatrix <- table(Predicted, Actual)
  ConfusionMatrix
  
  accuracy <- sum(diag(ConfusionMatrix)) / sum(ConfusionMatrix)
  
  intercept <- glm.fit$coefficients[1]
  bright_coef <- glm.fit$coefficients[2]
  rhomb_coef <- glm.fit$coefficients[3]
  
  results <- c(seed, intercept, bright_coef, rhomb_coef, accuracy)
  
  biglist <- c(biglist, results)
  seed = seed + 1
}

#out of for loop

bootstrap_result_WT_average_and_QRFP <- matrix(unlist(biglist), ncol = 5, byrow = TRUE)
colnames(bootstrap_result_WT_average_and_QRFP) <- c("Seed", "Intercept", "Brightness_Coeff","RhombQRFP_Coeff", "Accuracy")

bootstrap_result_WT_average_and_QRFP <- as_tibble(bootstrap_result_WT_average_and_QRFP)

avg_accuracy_WT_average_and_QRFP <- mean(bootstrap_result_WT_average_and_QRFP$Accuracy)
#avg_accuracy_WT_average_and_QRFP
sd_accuracy_WT_average_and_QRFP <- sd(bootstrap_result_WT_average_and_QRFP$Accuracy)
#sd_accuracy_WT_average_and_QRFP
```

#### 6.4.4.2 Multiple Logistic Regression with Avg Brightness + Rhomb QRFP, hets

Bootstrapping on het larvae…

```
het_average_and_QRFP <- average_and_QRFP %>%
  filter(geno == "het") 

het_average_and_QRFP$pheno <- as.factor(het_average_and_QRFP$pheno)

#bootstrapping logistic regression
seed = 0
biglist <- list()
sample_size = ceiling(0.5*nrow(het_average_and_QRFP))

# within for loop
for (seed in 1:100) {
  set.seed(seed)
  
  # randomly split data 

  chosen = sample(seq_len(nrow(het_average_and_QRFP)),size = sample_size)
  glm.training = het_average_and_QRFP[chosen,]
  glm.testing = het_average_and_QRFP[-chosen,]
  
  #multiple regression this time!
  glm.fit <- glm(pheno ~ average_signal + rhombQRFP, data = glm.training, family = binomial) 
  
  glm.probs <- predict(glm.fit,
                       newdata = glm.testing,
                       type = "response")
  
  # contrasts(glm.testing$pheno)# tells me which value has been assigned to which phenotype
  
  Predicted <- ifelse(glm.probs > 0.5, "SLC", "LLC")
  
  Actual <- glm.testing$pheno
  
  ConfusionMatrix <- table(Predicted, Actual)
  ConfusionMatrix
  
  accuracy <- sum(diag(ConfusionMatrix)) / sum(ConfusionMatrix)
  
  intercept <- glm.fit$coefficients[1]
  bright_coef <- glm.fit$coefficients[2]
  rhomb_coef <- glm.fit$coefficients[3]
  
  results <- c(seed, intercept, bright_coef, rhomb_coef, accuracy)
  
  biglist <- c(biglist, results)
  seed = seed + 1
}

#out of for loop

bootstrap_result_het_average_and_QRFP <- matrix(unlist(biglist), ncol = 5, byrow = TRUE)
colnames(bootstrap_result_het_average_and_QRFP) <- c("Seed", "Intercept", "Brightness_Coeff","RhombQRFP_Coeff", "Accuracy")

bootstrap_result_het_average_and_QRFP <- as_tibble(bootstrap_result_het_average_and_QRFP)

avg_accuracy_het_average_and_QRFP <- mean(bootstrap_result_het_average_and_QRFP$Accuracy)
#avg_accuracy_het_average_and_QRFP
sd_accuracy_het_average_and_QRFP <- sd(bootstrap_result_het_average_and_QRFP$Accuracy)
#sd_accuracy_het_average_and_QRFP
```

#### 6.4.4.3 Multiple Logistic Regression with Avg Brightness + Rhomb QRFP, mutants

Bootstrapping on mutant larvae…

```
mut_average_and_QRFP <- average_and_QRFP %>%
  filter(geno == "mut") %>%
    filter(!(collected == "210606")) %>%
      filter(!(collected == "210613")) 

mut_average_and_QRFP$pheno <- as.factor(mut_average_and_QRFP$pheno)

#bootstrapping logistic regression
seed = 0
biglist <- list()
sample_size = ceiling(0.5*nrow(mut_average_and_QRFP))

# within for loop
for (seed in 1:100) {
  set.seed(seed)
  
  # randomly split data 

  chosen = sample(seq_len(nrow(mut_average_and_QRFP)),size = sample_size)
  glm.training = mut_average_and_QRFP[chosen,]
  glm.testing = mut_average_and_QRFP[-chosen,]
  
  #multiple regression this time!
  glm.fit <- glm(pheno ~ average_signal + rhombQRFP, data = glm.training, family = binomial) 
  
  glm.probs <- predict(glm.fit,
                       newdata = glm.testing,
                       type = "response")
  
  # contrasts(glm.testing$pheno)# tells me which value has been assigned to which phenotype
  
  Predicted <- ifelse(glm.probs > 0.5, "SLC", "LLC")
  
  Actual <- glm.testing$pheno
  
  ConfusionMatrix <- table(Predicted, Actual)
  ConfusionMatrix
  
  accuracy <- sum(diag(ConfusionMatrix)) / sum(ConfusionMatrix)
  
  intercept <- glm.fit$coefficients[1]
  bright_coef <- glm.fit$coefficients[2]
  rhomb_coef <- glm.fit$coefficients[3]
  
  results <- c(seed, intercept, bright_coef, rhomb_coef, accuracy)
  
  biglist <- c(biglist, results)
  seed = seed + 1
}

#out of for loop

bootstrap_result_mut_average_and_QRFP <- matrix(unlist(biglist), ncol = 5, byrow = TRUE)
colnames(bootstrap_result_mut_average_and_QRFP) <- c("Seed", "Intercept", "Brightness_Coeff","RhombQRFP_Coeff", "Accuracy")

bootstrap_result_mut_average_and_QRFP <- as_tibble(bootstrap_result_mut_average_and_QRFP)

avg_accuracy_mut_average_and_QRFP <- mean(bootstrap_result_mut_average_and_QRFP$Accuracy)
#avg_accuracy_mut_average_and_QRFP
sd_accuracy_mut_average_and_QRFP <- sd(bootstrap_result_mut_average_and_QRFP$Accuracy)
#sd_accuracy_mut_average_and_QRFP
```

### 6.4.5 All logistic regression accuracy results

```
#Assemble all Logistic Regression Data
logistic_QRFP_and_average_accuracy <- c(avg_accuracy_WT_average_and_QRFP, 
                                        avg_accuracy_het_average_and_QRFP, 
                                        avg_accuracy_mut_average_and_QRFP)

logistic_QRFP_and_average_sd <- c(sd_accuracy_WT_average_and_QRFP, 
                      sd_accuracy_het_average_and_QRFP, 
                      sd_accuracy_mut_average_and_QRFP)

model_QRFP_avg <- c("Average + DCR6", "Average + DCR6", "Average + DCR6")

logistic_QRFP_and_average_accuracy_tibble <- tibble(genotypes_longhand, 
                               model_QRFP_avg, 
                               logistic_QRFP_and_average_accuracy,
                               logistic_QRFP_and_average_sd)
colnames(logistic_QRFP_and_average_accuracy_tibble) = (c("Genotype","Model","Accuracy","StDev"))

gt_QRFP_and_average_table <- gt(logistic_QRFP_and_average_accuracy_tibble) 
gt_QRFP_and_average_table
```

| Genotype | Model | Accuracy | StDev |
| --- | --- | --- | --- |
| +/+ | Average + DCR6 | 0.6932000 | 0.13430615 |
| +/- | Average + DCR6 | 0.4730769 | 0.07246652 |
| -/- | Average + DCR6 | 0.6680000 | 0.20542147 |

When we graph the same data, we see:

```
full_logistic_tibble <- rbind(logistic_accuracy_tibble,
                          logistic_QRFP_tibble,
                          logistic_QRFP_and_average_accuracy_tibble) %>%
  mutate(Genotype = as.factor(Genotype)) %>%
  #mutate(Genotype = arrange(Genotype)) %>%          
  mutate(Model = as.factor(Model)) %>%
 # mutate(Model = fct_relevel(Model, c("Whole-brain average", "DCR6", "Average + DCR6"))) %>%
  mutate(Accuracy = (Accuracy*100)) %>% #percentages
  mutate("StDev" = (StDev*100)) %>% #percentages
  mutate("SEM" = StDev/10) %>% #because I ran 100 simulations and sqrt(100) = 10
  mutate("SEM" = as.numeric(SEM)) %>%
  mutate("errmin" = (Accuracy - SEM)) %>%
  mutate("errmax" = Accuracy + SEM)

#write_csv(full_logistic_tibble, "Bootstrap_Accuracy_Comparisons_220204.csv")

#Plot all data

logistic_graph_FacetModel <- ggplot(full_logistic_tibble, aes(x = Genotype, y= Accuracy, fill = Genotype)) +
  ggtitle(corr_title) +
  geom_bar(stat = "identity") +
  geom_errorbar(aes(x = Genotype, ymin = (errmin), ymax = (errmax)),
                width=0.2, size=1, color="grey20") +
  scale_fill_manual(values = c("#003fff", "#80B655", "#B53737"), 
                               labels = c("+/+", "+/p190","p190/p190")) +
  labs(fill = "CaSR Genotype") +
  facet_wrap(~Model) +
  scale_y_continuous("Percent of Test Brains\n Categorized Correctly",
                     breaks = c(0,25,50,75,100),
                     limits = c(0,110),
                     expand = c(0,0)) +
  scale_x_discrete("Variables Used in Logistic Regression Model") +
    theme(
        plot.title = element_blank(),
      #  axis.text.x = element_text(color = "black", size = 8, angle = 45, hjust = 1),
        axis.text.x = element_blank(),
        axis.text.y = element_text(color = "black", size = 8),
        axis.ticks = element_blank(),
        panel.grid.major.y = element_line(colour = "grey80"),
        panel.grid.major.x = element_blank(),
        panel.background = element_rect(fill = NA),
      #  legend.position = "none",
        axis.title.y = element_text(size=10, face = "bold"),
        axis.title.x = element_text(size=10, face = "bold"),
        strip.text = element_text(size = 10))

logistic_graph_FacetModel
```

An alternative way of formatting the graph above can be displayed by changing `eval = FALSE` in the code chunk below to `eval = TRUE.`

```
# A different version of the same graph

logistic_graph_FacetGeno <- ggplot(full_logistic_tibble, aes(x = Model, y= Accuracy, fill = Model)) +
  ggtitle(corr_title) +
  geom_bar(stat = "identity") +
  geom_errorbar(aes(x = Model, ymin = (errmin), ymax = (errmax)),
                width=0.2, size=1, color="grey20") +
  scale_fill_manual(values = c("#238A31", "#80B655", "#ACC674"),
                               labels = c("Avg Over\nWhole Brain", "DCR6", "Avg. + DCR6")) +
  labs(fill = "Variables Used\nFor Model") +
  facet_wrap(~Genotype) +
  scale_y_continuous("Percent of Test Brains\n Categorized Correctly",
                     breaks = c(0,25,50,75,100),
                     limits = c(0,110),
                     expand = c(0,0)) +
  scale_x_discrete("Variables Used in Logistic Regression Model") +
    theme(
        plot.title = element_blank(),
      #  axis.text.x = element_text(color = "black", size = 8, angle = 45, hjust = 1),
        axis.text.x = element_blank(),
        axis.text.y = element_text(color = "black", size = 8),
        axis.ticks = element_blank(),
        panel.grid.major.y = element_line(colour = "grey80"),
        panel.grid.major.x = element_blank(),
        panel.background = element_rect(fill = NA),
      #  legend.position = "none",
        axis.title.y = element_text(size=10, face = "bold"),
        axis.title.x = element_text(size=10, face = "bold"),
        strip.text = element_text(size = 10))

logistic_graph_FacetGeno

# ggsave("Avg_vs_DCR6_SEMErrBars.png",
#        device = png, 
#        units = "px", 
#        width = 1500,
#        height = 1125,
#        dpi = 300)

# ggarrange(corr_by_geno, logistic_graph, ncol = 1, nrow =2, labels = c("A", "B"))
# 
# ggsave("Avg_vs_DCR6_Predictions_FacetModel.png",
#        device = png, 
#        units = "px", 
#        width = 1500,
#        height = 1125,
#        dpi = 300)
```

# 7 Identifying alternative candidate brain regions

We were able to validate the importance of the DCR6 / Rhombencephalon Cluster QRFP by overexpressing CaSR specifically in the region and recapitulating the CaSR gain-of-function phenotype + rescuing the CaSR loss-of-function mutant phenotype. However, if we had not been able to do this, we would have needed to find other brain regions where CaSR expression strongly correlates with phenotype. In this section, we give examples of two approaches to find alternative regions. The first is simply to determine which regions are most highly correlated with your initial candidate region. The second is to bootstrap the LASSO regression and see if other patterns emerge from your data.

## 7.1 Correlational analysis: which other regions correlate with the DCR6?

First, we create a correlation matrix for the whole brain.

```
#### turn it into a matrix, remove metadata ####
NormedMatrix <- as.matrix(normed)
rownames(NormedMatrix) <- NormedMatrix[,1] #turns uniqueID for each fish into rownames
NormedMatrix <- NormedMatrix[, -(1:7)] #gets rid of metadata columns

NormedMatrixNumeric <- matrix(as.numeric(unlist(NormedMatrix)),nrow=nrow(NormedMatrix))
rownames(NormedMatrixNumeric) <- rownames(NormedMatrix)
colnames(NormedMatrixNumeric) <- colnames(NormedMatrix)

#### Make correlation matrix of whole-brain expression data ####
corr_wholebrain <- cor(NormedMatrixNumeric)
plain_corr_plot <- corrplot(corr_wholebrain, method = 'color', tl.pos = "n", order = 'hclust')
```

```
#### Extract correlations of a single region ####
corr_wholebrain_tib <- as_tibble(corr_wholebrain)

#write_csv(corr_wholebrain_tib, "PearsonCorrelation_AllBrainRegions.csv")
```

For a version of the plot above with non-significant correlations excluded, set `eval= TRUE` for the code chunk below.

```
testRes = cor.mtest(NormedMatrixNumeric, conf.level = 0.95)

plain_corr_plot_no_insig <- corrplot(corr_wholebrain, method = 'color', tl.pos = "n", order = 'hclust',
                                    p.mat = testRes$p, insig='blank', number.cex = 0.8)
```

### 7.1.1 Correlations of the DCR6 only

This code chunk contains a function, `exportStrongestCorrelatedRegions()`, that will output a .csv file that can be supplied to the `CustomBrainRegionStack()` function in Matlab in order to make a 3D visualization of the brain regions that most highly correlate with your ROI.

```
corr_wholebrain_tib$region <- regionNames
rhomb_QRFP_corr <- corr_wholebrain_tib %>%
  select(region, rhombencephalon_qrfp_neuron_cluster_sparse)
rhomb_QRFP_corr_ranked <- rhomb_QRFP_corr %>%
  arrange(desc(rhombencephalon_qrfp_neuron_cluster_sparse)) %>%
  filter(region != "rhombencephalon_qrfp_neuron_cluster_sparse")

rhomb_QRFP_corr_fx <- exportStrongestCorrelatedRegions(corr_object = corr_wholebrain,
                                    brain_region = "rhombencephalon_qrfp_neuron_cluster_sparse",
                                    num2show = 12,
                                    filtered = "rhomb7")

#Uncomment the line below to run function that will export a .csv:

#rhomb_QRFP_corr_fx
```

#### 7.1.1.1 Table of the regions most and least correlated with the DCR6

```
most_corr_regions_QRFP <- gt(rhomb_QRFP_corr_fx)

most_corr_regions_QRFP
```

| ROIname | corr |
| --- | --- |
| rhombencephalon\_vglut2\_cluster\_2 | 0.89572889 |
| rhombencephalon\_rhombomere\_6 | 0.89335614 |
| rhombencephalon | 0.87400309 |
| rhombencephalon\_gad1b\_cluster\_14 | 0.86509444 |
| rhombencephalon\_cerebellum | 0.85622294 |
| rhombencephalon\_corpus\_cerebelli | 0.84889977 |
| rhombencephalon\_rhombomere\_1 | 0.84866634 |
| rhombencephalon\_6\_7f\_dhcrt\_r\_gal4\_stripe\_2 | 0.84504665 |
| rhombencephalon\_olig2\_enriched\_areas\_in\_cerebellum | 0.84420743 |
| rhombencephalon\_s1181t\_cluster | 0.84329638 |
| diencephalon\_left\_habenula\_vglut2\_cluster | 0.83643353 |
| rhombencephalon\_cerebellum\_gad1b\_enriched\_areas | 0.83239943 |
| rhombencephalon\_mi\_r1 | 0.08436974 |
| diencephalon\_dopaminergic\_cluster\_4\_5\_posterior\_tuberculum\_and\_hypothalamus | 0.08431436 |
| rhombencephalon\_ro\_v3 | 0.07968660 |
| diencephalon\_hypothalamus\_vglut2\_cluster\_6 | 0.07958511 |
| diencephalon\_hypothalamus\_qrfp\_neuron\_cluster | 0.07176137 |
| diencephalon\_otpb\_cluster\_1 | 0.06719860 |
| diencephalon\_hypothalamus\_gad1b\_cluster\_1 | 0.05793189 |
| diencephalon\_migrated\_posterior\_tubercular\_area\_m2 | 0.05767190 |
| diencephalon\_postoptic\_commissure | 0.05413082 |
| diencephalon\_hypothalamus\_vglut2\_cluster\_2 | 0.05313499 |
| diencephalon\_retinal\_arborization\_field\_1\_af1\_approximate\_location | 0.05158826 |
| ganglia\_lateral\_line\_neuromast\_o1 | 0.02216119 |

### 7.1.2 Bootstrapping LASSO regression

When we use the whole dataset to train our model, as we did above, we lose the opportunity to assess how accurately our model can predict data that was not used to train the model. To do this, I will bootstrap the model, as I did above in the univariate analysis.

```
#### a different method for lasso regression using testing and training sets to estimate accuracy
# based on: http://www.science.smith.edu/~jcrouser/SDS293/labs/lab10-r.html

seed = 0
biglist <- list()

for (seed in 1:100) {
  set.seed(seed)
  train_lasso <- WTNumeric %>%
    sample_frac(0.5)  #matches what I did for univariate logistic regression, where half of the data was used fur training and half for testing

  test_lasso = WTNumeric %>%
    setdiff(train_lasso)

  x_train = model.matrix(pheno~., train_lasso)[,-1]
  x_test = model.matrix(pheno~., test_lasso)[,-1]

  y_train = train_lasso %>%
    select(pheno) %>%
    unlist() %>%
    as.numeric()

  y_test = test_lasso %>%
    select(pheno) %>%
    unlist() %>%
    as.numeric()

  lasso_mod = glmnet(x_train,
                     y_train,
                     alpha = 1,
                     lambda = avg_bestlam) # Fit lasso model on training data

  # accuracy for training data
  lasso_pred_train = predict(lasso_mod, s = avg_bestlam, newx = x_train) # Use best lambda to predict test data
  trainMSE <- mean((lasso_pred_train - y_train)^2) # Calculate test MSE

  Predicted <- ifelse(lasso_pred_train > 0.5, "SLC", "LLC")

  Actual <- ifelse(y_train > 0.5, "SLC", "LLC") #this happens to work because I coded SLC as 1 and LLC as 0.

  TrainConfusionMatrix <- table(Predicted, Actual)

  train_accuracy_lasso <- sum(diag(TrainConfusionMatrix)) / sum(TrainConfusionMatrix)

  # accuracy_lasso for test data
  lasso_pred_test = predict(lasso_mod, s = avg_bestlam, newx = x_test) # Use best lambda to predict test data
  testMSE <- mean((lasso_pred_test - y_test)^2) # Calculate test MSE

  PredictedTest <- ifelse(lasso_pred_test > 0.5, "SLC", "LLC")

  ActualTest <- ifelse(y_test > 0.5, "SLC", "LLC") #this happens to work because I coded SLC as 1 and LLC as 0.

  TestConfusionMatrix <- table(PredictedTest, ActualTest)

  test_accuracy_lasso <- sum(diag(TestConfusionMatrix)) / sum(TestConfusionMatrix)

  # add to the table

  results <- c(seed, train_accuracy_lasso, trainMSE, test_accuracy_lasso, testMSE)

  biglist <- c(biglist, results)
  seed = seed + 1
}

bootstrap_result <- matrix(unlist(biglist), ncol = 5, byrow = TRUE)
colnames(bootstrap_result) <- c("seed", "training_accuracy_lasso", "training_MSE", "testing_accuracy_lasso", "testingMSE")

bootstrap_result <- as_tibble(bootstrap_result) %>%
  mutate("Quality" = case_when(
    ((training_accuracy_lasso>0.5 & testing_accuracy_lasso >0.5)~ "Accurate"),
    ((training_accuracy_lasso<0.5 & testing_accuracy_lasso <0.5)~ "Inaccurate"),
    (TRUE ~ "Other") 
  )) %>%
  mutate(Quality = as.factor(Quality))

#write_csv(bootstrap_result, file = "BimodalBootstrapAccuracy.csv")

position <- position_jitter(0.02, 0.02)

bimodal_accuracy_plot <- ggplot(bootstrap_result, aes(x = training_accuracy_lasso, 
                                                      y = testing_accuracy_lasso,
                                                      color = Quality)) +
  geom_point(shape = 1, size = 2, position = position) +
  scale_x_continuous(limits = c(0.0,1.01), expand = c(0,0)) +
  scale_y_continuous(limits = c(0,1.01), expand = c(0,0)) +
  scale_color_manual(values = c("#009999", "#7573b6", "black")) +
  coord_fixed() +
#  geom_rect(aes(xmin = .5, xmax = 0.99, ymin = .5, ymax =0.99),
   #         color = "#90d2c3", alpha = 0.000001, size = 0.5) +
#  geom_rect(aes(xmin = 0.01, xmax = .5, ymin = 0.01, ymax = .5),
     #       color = "#7573b6", alpha = 0.000001, size = 0.5) +
#  ggtitle("Accuracy of LASSO Models") +
#  labs(subtitle = "Bootstrapping on WT larvae") +
  xlab("Accuracy Categorizing Training Set") +
  ylab("Accuracy Categorizing Testing Set") +
  theme(
    text=element_text(family="Arial"),
    axis.text = element_text(size = 8),
    axis.title = element_text(size = 10),
    panel.background = element_blank(),
    axis.line = element_line(color = "grey40"),
    panel.grid.major.y = element_blank(),
    panel.grid.major.x = element_blank(),
    strip.text.x = element_text(size = 10)
  )

 bimodal_accuracy_plot
```

```
# 
# ggsave("Bimodal_Accuracy_220118.png",
#     device = "png",
#      width = 4800,
#      height = 4800,
#      units = ('px'),
#      dpi = 1200)
# 
# ggsave("Bimodal_Accuracy_220118.pdf",
#     device = "png",
#      width = 4800,
#      height = 4800,
#      units = ('px'),
#      dpi = 1200)
#
```

#### 7.1.2.1 LASSO regression: intrepreting bootstrapped analysis

There appears to be a bimodal distribution of the quality of the LASSO models constructed! The ones in green give results much better than chance for both training and testing, but the ones in purple are worse than chance in both cases. What is the difference between an accurate and an inaccurate model? In order to reconstruct this, first I will identify the individual random seeds that produced accurate or inaccurate models. Then I will examine the characteristics of models that produced good results verus models that produced bad results.

```
#### find the good models

good_models <- bootstrap_result %>%
  filter(testing_accuracy_lasso > 0.5 & training_accuracy_lasso > 0.5)

print(paste("Found", nrow(good_models), "good seeds."))
```

```
## [1] "Found 35 good seeds."
```

```
good_model_seeds <- good_models$seed

mat <- matrix(nrow = 252, ncol = 1)
good_coeff_tib <- as_tibble(mat)
seedlist <- c()

for(seed in good_model_seeds) {

  set.seed(seed)
  train_lasso <- WTNumeric %>%
    sample_frac(0.5)

  test_lasso = WTNumeric %>%
    setdiff(train_lasso)

  x_train = model.matrix(pheno~., train_lasso)[,-1]
  x_test = model.matrix(pheno~., test_lasso)[,-1]

  y_train = train_lasso %>%
    select(pheno) %>%
    unlist() %>%
    as.numeric()

  y_test = test_lasso %>%
    select(pheno) %>%
    unlist() %>%
    as.numeric()

  lasso_mod = glmnet(x_train,
                     y_train,
                     alpha = 1,
                     lambda = avg_bestlam) # Fit lasso model on training data

  lasso.coef = predict(lasso_mod, type = "coefficients", s = avg_bestlam)

  lasso.coef <- as_tibble(as.matrix(lasso.coef))

  good_coeff_tib <- cbind(good_coeff_tib, lasso.coef)
}

# is.na(good_coeff_tib) <- good_coeff_tib==0 #replaces 0s with NAs
colnames(good_coeff_tib) <- c("SeedNum",good_model_seeds) #there's an extra column in here that needs a name
good_coeff_tib <- as_tibble(good_coeff_tib)

#trasnposing so I can do analysis with regions as columns
good_transposed <- as_tibble(t(as.matrix(good_coeff_tib))) 
colnames(good_transposed) <- c("Intercept",regionNames) # there's an extra value here, which is what the lasso regression has identified as the Intercept / base probability that a brain will be SLC or LLC. Intercept is related to the proportion of SLC vs LLC brains in the training dataset, so I don't pay it much attention

## Summarizing by seed 
  #- for each seed that used Rhomb QRFP region, what was the value? how many other regions did it use?

# was rhombencephalon QRFP cluster used as a coefficient in this model?
good_QRFP_yes <- case_when((good_transposed$rhombencephalon_qrfp_neuron_cluster_sparse == 0) ~ 0,
                           (good_transposed$rhombencephalon_qrfp_neuron_cluster_sparse != 0) ~ 1) 

#how many other nonzero coefficients did this model assign?
good_counts_by_seed <- rowSums(good_transposed != 0, na.rm = TRUE)

#making it a tibble
good_summary_by_seed <- tibble(
  "seed" = colnames(good_coeff_tib),
  "counts_by_seed" = good_counts_by_seed,
  "QRFP_yes" = good_QRFP_yes,
  "signal_in_QRFP" = good_transposed$rhombencephalon_qrfp_neuron_cluster_sparse
)

good_summary_by_seed <- good_summary_by_seed[-c(1), ]  # dropping extraneous row in tibble

good_summary_by_seed <- good_summary_by_seed %>%
  mutate("seed_category" = "Accurate") %>% # for later merging with bad seed summary
  mutate("non_RhombQRFP_coeffs_count" = counts_by_seed - QRFP_yes) #how many coefficients OTHER THAN Rhombencephalon QRFP Cluster Sparse did the model use?

# Summarizing by region
 # - what was the average weight of each region? how many times was each region used in good models?

good_means_by_region <- colMeans(good_transposed, na.rm = TRUE) # average value of coefficient of each region 

good_counts_by_region <- colSums(good_transposed != 0, na.rm = TRUE) # number of times a given region is assigned a coefficient 

good_summary_by_region <- tibble(
  "region" = c("Intercept",regionNames),
  "mean_coefficient" = good_means_by_region,
  "count_included" = good_counts_by_region,
  "seed_category" = "Accurate"
)

good_summary_by_region <- good_summary_by_region %>%
  dplyr::mutate(percent_models_included = count_included / nrow(good_models))

#individual datapoints on all regions
good_transposed$seed_num <- c("seed_num", good_model_seeds)

good_datapoints <- good_transposed[-c(1), ] %>% # dropping extraneous row in tibble
  pivot_longer(!seed_num, names_to = "Region", values_to = "Coefficient_Weight") %>%
  filter(Coefficient_Weight != 0) %>%
  filter(Region != "Intercept") %>%
  mutate("seed_category" = "Accurate")

# write.csv(good_coeff_tib, "LassoRegression_OnWTs_GoodSeeds_210728.csv")

#### And now find the bad models
bad_models <- bootstrap_result %>%
  filter(testing_accuracy_lasso < 0.5 & training_accuracy_lasso < 0.5)

print(paste("Found", nrow(bad_models), "bad seeds."))
```

```
## [1] "Found 55 bad seeds."
```

```
bad_model_seeds <- bad_models$seed

mat <- matrix(nrow = 252, ncol = 1)
bad_coeff_tib <- as_tibble(mat)
seedlist <- c()

for(seed in bad_model_seeds) {

  set.seed(seed)
  train_lasso <- WTNumeric %>%
    sample_frac(0.5)

  test_lasso = WTNumeric %>%
    setdiff(train_lasso)

  x_train = model.matrix(pheno~., train_lasso)[,-1]
  x_test = model.matrix(pheno~., test_lasso)[,-1]

  y_train = train_lasso %>%
    select(pheno) %>%
    unlist() %>%
    as.numeric()

  y_test = test_lasso %>%
    select(pheno) %>%
    unlist() %>%
    as.numeric()

  lasso_mod = glmnet(x_train,
                     y_train,
                     alpha = 1,
                     lambda = avg_bestlam) # Fit lasso model on training data

  lasso.coef = predict(lasso_mod, type = "coefficients", s = avg_bestlam)

  lasso.coef <- as_tibble(as.matrix(lasso.coef))

  bad_coeff_tib <- cbind(bad_coeff_tib, lasso.coef)
}

# is.na(bad_coeff_tib) <- bad_coeff_tib==0 #replaces 0s with NAs
colnames(bad_coeff_tib) <- c("SeedNum",bad_model_seeds) #there's an extra column in here that needs a name
bad_coeff_tib <- as_tibble(bad_coeff_tib)

#trasnposing so I can do analysis with regions as columns
bad_transposed <- as_tibble(t(as.matrix(bad_coeff_tib))) 
colnames(bad_transposed) <- c("Intercept",regionNames) # there's an extra value here, which is what the lasso regression has identified as the Intercept / base probability that a brain will be SLC or LLC. Intercept is related to the proportion of SLC vs LLC brains in the training dataset, so I don't pay it much attention

## Summarizing by seed 
  #- for each seed that used Rhomb QRFP region, what was the value? how many other regions did it use?

# was rhombencephalon QRFP cluster used as a coefficient in this model?
bad_QRFP_yes <- case_when((bad_transposed$rhombencephalon_qrfp_neuron_cluster_sparse == 0) ~ 0,
                           (bad_transposed$rhombencephalon_qrfp_neuron_cluster_sparse != 0) ~ 1) 

#how many other nonzero coefficients did this model assign?
bad_counts_by_seed <- rowSums(bad_transposed != 0, na.rm = TRUE)

#making it a tibble
bad_summary_by_seed <- tibble(
  "seed" = colnames(bad_coeff_tib),
  "counts_by_seed" = bad_counts_by_seed,
  "QRFP_yes" = bad_QRFP_yes,
  "signal_in_QRFP" = bad_transposed$rhombencephalon_qrfp_neuron_cluster_sparse
)

bad_summary_by_seed <- bad_summary_by_seed[-c(1), ]  # dropping extraneous row in tibble

bad_summary_by_seed <- bad_summary_by_seed %>%
  mutate("seed_category" = "Inaccurate") %>% # for later merging with bad seed summary
  mutate("non_RhombQRFP_coeffs_count" = counts_by_seed - QRFP_yes) #how many coefficients OTHER THAN Rhombencephalon QRFP Cluster Sparse did the model use?

# Summarizing by region
 # - what was the average weight of each region? how many times was each region used in bad models?

bad_means_by_region <- colMeans(bad_transposed, na.rm = TRUE) # average value of coefficient of each region 

bad_counts_by_region <- colSums(bad_transposed != 0, na.rm = TRUE) # number of times a given region is assigned a coefficient 

bad_summary_by_region <- tibble(
  "region" = c("Intercept",regionNames),
  "mean_coefficient" = bad_means_by_region,
  "count_included" = bad_counts_by_region,
  "seed_category" = "Inaccurate"
)

bad_summary_by_region <- bad_summary_by_region %>%
  dplyr::mutate(percent_models_included = count_included / nrow(bad_models))

#individual datapoints on all regions
#individual datapoints on all regions
bad_transposed$seed_num <- c("seed_num", bad_model_seeds)

bad_datapoints <- bad_transposed[-c(1), ] %>% # dropping extraneous row in tibble
  pivot_longer(!seed_num, names_to = "Region", values_to = "Coefficient_Weight") %>%
  filter(Coefficient_Weight != 0) %>%
  filter(Region != "Intercept") %>%
  mutate("seed_category" = "Inaccurate")
```

#### 7.1.2.2 Graphing traits of models made with accurate vs. inaccurate seeds

The Rhombencephalon QRFP cluster - sparse is the most commonly-used coefficient in both good and bad models. If this brain region is the only one that matters for phenotype, then good models should use only this region, and bad models might use other regions. Alternatively, if other regions contribute, then good models might be more likely to employ multiple regions in their models, while bad models might over-rely on the Rhombencephalon QRFP Cluster - Sparse.

From this analysis, it seems like good models are more likely to employ more regions than bad models, suggesting that CaSR expression levels in multiple regions – with the DCR6 being by far the most prominent – may influence phenotype.

```
# time to bring my summaries together
## start with seed
total_summary_by_seed <- rbind(bad_summary_by_seed, good_summary_by_seed)

position <- position_jitter(0.1, 0)

seeds_graph_QRFP <- ggplot(total_summary_by_seed, aes(x= non_RhombQRFP_coeffs_count,
                                                 y= signal_in_QRFP, 
                                                 color = seed_category)) +
    geom_point(size = 1, position = position) +
    facet_wrap(~seed_category, nrow = 2) +
    scale_x_continuous(limits = c(0,8), expand = c(0,0)) +
    scale_y_continuous(limits = c(0,0.12), expand = c(0,0)) +
    scale_color_manual(name = "Seed Category", values = c("#006666","#7573b6")) +
 #   ggtitle("Characterizing Accurate vs. Inaccurate Lasso Logistic Regression Models") +
  #  labs(subtitle = "Bootstrapping on WT larvae") +
    xlab("Number of Other Brain Regions Incorporated Into Model") +
    ylab("Weight of 'Rhombencephalon QRFP\nCluster Sparse' Coefficient") +
    theme(
      text=element_text(family="Arial"),
      panel.background = element_blank(),
      axis.line = element_line(color = "grey40"),
      axis.text = element_text(size = 8),
      axis.title = element_text(size = 10),
      panel.grid.major.y = element_line(color = "grey80"),
      strip.text.x = element_text(size = 10),
      legend.title = element_text(size = 10),
      legend.text = element_text(size = 8),
      plot.margin = unit(c(0.1,0.1,0.1,0.1), "cm")
  )

seeds_graph_QRFP
```

```
# ggsave("Seeds_210806.png",       
#        device = "png",
#        width = 8, 
#        height = 5, 
#        units = ('in'),
#        dpi = 300)
# 
# ggsave("Seeds_210806.tif",
#       device = "tiff",
#        width = 8, 
#        height = 5, 
#        units = ('in'),
#        dpi = 300)
# 
# write_csv(total_summary_by_seed, "210809_SeedSummary_OnlyRhombQRFP.csv")
```

#### 7.1.2.3 Graphing the number of times a region appears in accurate vs. inaccurate models

Are there any regions that feature prominently in good models, but not bad models? Maybe we can identify other regions in which CaSR expression level drives changes in phenotype.

```
## now by region
total_summary_by_region <- rbind(bad_summary_by_region, good_summary_by_region) 
total_summary_by_region <- total_summary_by_region %>%
  filter(count_included > 1) %>%
  arrange(desc(percent_models_included))

region_counts_graph <- ggplot(total_summary_by_region, aes(
                  x= reorder(region, percent_models_included), 
                  y= percent_models_included, 
                  fill = seed_category)) +
    coord_flip() +
    facet_wrap(~seed_category) +
    geom_col(size = 2, position = "dodge") +
    scale_y_continuous(limits = c(0,1), expand = c(0,0), breaks = c(0,0.5, 1)) +
    scale_fill_manual(name = "Seed Category", values = c("#006666","#7573b6")) +
    ggtitle("Percent of Models Using Coefficient") +
    labs(subtitle = "Bootstrapping on WT larvae,\nRegions Observed in >1 Model") +
    xlab("Region") +
    ylab("Percent of Models Using Region") +
    labs(fill = "Seed Category") +
    theme(
      text=element_text(family="Arial"),
      panel.background = element_blank(),
      panel.spacing = unit(1, "lines"),
      axis.line = element_line(color = "grey40"),
      panel.grid.major.y = element_line(color = "grey80"),
      strip.text.x = element_text(size = 8)
  )

region_counts_graph
```

```
# ggsave("RegionCounts_210806.png",       
#        device = "png",
#        width = 8, 
#        height = 5, 
#        units = ('in'),
#        dpi = 600)
# 
# ggsave("RegionCounts_210806.tif",
#       device = "tiff",
#        width = 8, 
#        height = 5, 
#        units = ('in'),
#        dpi = 600)
# 
#write_csv(total_summary_by_region, "RegionPrevalence_SeedType.csv")
```

#### 7.1.2.4 Which brain regions with POSITIVE coefficients (in accurate models) are also positively correlated with DCR6?

Of course, if a region appears often among the “good models” one possibility is simply that it highly correlates with and therefore proxies for signal in the DCR6. To check for this possibility, we cross-referenced the list of regions that appeared commonly among “good models” with the top 10 regions that most highly correlate with the DCR6.

```
#Making an object that contains Good Seed data-- which regions were used in Good Seeds and how often
#Dividing up by whether regions were positively or negatively weighted in LASSO

good_seed_regions_pos <- good_summary_by_region %>%
  filter(count_included > 1) %>%  # don't want extremely infrequent regions cluttering graph
  filter(mean_coefficient > 0) %>% #because only positively weighted regions
  mutate(percent_models_included = percent_models_included*100) %>%
  arrange(desc(percent_models_included)) %>%
  filter(region != "Intercept")

#load in file with different forms of brain region names
brain_regions_translator <- read_xlsx("293_BrainRegions_Translator.xlsx")

#graph with long regions names is very hard to read--> adding in short region names with left_join
good_seed_regions_pos_names <- left_join(good_seed_regions_pos, brain_regions_translator, by = "region")

#Note: for list of regions positively correlated with DCR6, go to section "Correlational analysis: what other regions correlate with Rhombencephalon Cluster QRFP?"

good_seed_regions_pos_names$fill_colors <- c("#b55a4e", 
                                "#808080",
                                "#3fa0b5",
                                "#808080",
                                "#808080",
                                "#808080",
                                "#808080",
                                "#3fa0b5",
                                "#808080",
                                "#808080",
                                "#808080",
                                "#808080",
                                "#808080",
                                "#808080")


good_seed_regions_pos_graph <- ggplot(good_seed_regions_pos_names, aes(
                  x= reorder(short_spaces, percent_models_included), 
                  y= percent_models_included, 
                  fill = fill_colors)) +
    coord_flip() +
 #   facet_wrap(~seed_category) +
    geom_col(size = 2) +
    scale_y_continuous(limits = c(0,100), expand = c(0,0), breaks = c(0,50, 100)) +
    scale_fill_manual(name = "Region", 
                      values = c("#3fa0b5","#808080","#b55a4e"), 
                      labels = c("Most Corr", "Other", "DCR6")) +
    ggtitle("Positive Coefficients") +
   # labs(subtitle = "Bootstrapping on WT larvae,\nRegions Observed in >1 Model") +
    ylab("Percent of Models Using Region") +
    labs(fill = "Seed Category") +
    theme(
  #    text=element_text(family="Arial"),
      panel.background = element_blank(),
      panel.spacing = unit(1, "lines"),
      axis.line = element_line(color = "grey40"),
      panel.grid.major.y = element_blank(),
      axis.title.y = element_blank(),
      strip.text.x = element_text(size = 8)
  )
  
good_seed_regions_pos_graph
```

```
# ggsave("Good_Seeds_Pos_Coeffs_220207.pdf",
#        units = "px",
#        width = 4800,
#        height = 4800,
#        dpi = 1200)
# 
# ggsave("Good_Seeds_Pos_Coeffs_220207.png",
#        units = "px",
#        width = 4800,
#        height = 4800,
#        dpi = 1200)
```

#### 7.1.2.5 Which brain regions with NEGATIVE coefficients (in accurate models) are also positively correlated with DCR6?

Here we perform the same analysis as the above section, this time looking at regions which most often have *negative* coefficients in good models (e.g. high CaSR-EGFP signal correlates with LLC performance) and seeing if they are among the top 10 *least* correlated regions with the DCR6.

```
#Making an object that contains Good Seed data-- which regions were used in Good Seeds and how often
#Dividing up by whether regions were positively or negatively weighted in LASSO

good_seed_regions_neg <- good_summary_by_region %>%
  filter(count_included > 1) %>%  # don't want extremely infrequent regions cluttering graph
  filter(mean_coefficient < 0) %>% #because only positively weighted regions
  mutate(percent_models_included = percent_models_included*100) %>%
  arrange(desc(percent_models_included)) %>%
  filter(region != "Intercept")

#graph with long regions names is very hard to read--> adding in short region names with left_join
good_seed_regions_neg_names <- left_join(good_seed_regions_neg, brain_regions_translator, by = "region")

good_seed_regions_neg_names$fill_colors <- c("#50C878", 
                                "#808080",
                                "#808080",
                                "#808080",
                                "#808080",
                                "#808080",
                                "#808080")

  
good_seed_regions_neg_graph <- ggplot(good_seed_regions_neg_names, aes(
                  x= reorder(short_spaces, percent_models_included), 
                  y= percent_models_included, 
                  fill = fill_colors)) +
    coord_flip() +
    geom_col(size = 2) +
    scale_y_continuous(limits = c(0,100), expand = c(0,0), breaks = c(0,50, 100)) +
    scale_fill_manual(name = "Region", 
                      values = c("#50C878","#808080"), 
                      labels = c("Least Corr", "Other")) +
    ggtitle("Negative Coefficients") +
   # labs(subtitle = "Bootstrapping on WT larvae,\nRegions Observed in >1 Model") +
    xlab("Region") +
    ylab("Percent of Models Using Region") +
    labs(fill = "Seed Category") +
    theme(
  #    text=element_text(family="Arial"),
      panel.background = element_blank(),
      panel.spacing = unit(1, "lines"),
      axis.line = element_line(color = "grey40"),
      panel.grid.major.y = element_blank(),
      axis.title.y = element_blank(),
      strip.text.x = element_text(size = 8)
  )
  
good_seed_regions_neg_graph
```

```
# ggsave("Good_Seeds_Neg_Coeffs_220207.pdf",
#        units = "px",
#        width = 4800,
#        height = 3000,
#        dpi = 1200)
# 
# ggsave("Good_Seeds_Neg_Coeffs_220207.png",
#        units = "px",
#        width = 4800,
#        height = 3000,
#        dpi = 1200)
```

#### 7.1.2.6 Graphing coefficient weights of brain regions in accurate vs. inaccurate models

Finally we see if the “good models” or “bad models” weight these regions more. Interestingly, neither category of model weights the DCR6 very highly.

```
total_datapoints <- rbind(bad_datapoints, good_datapoints) 

total_datapoints_counts_filtered <- total_datapoints %>%
  group_by(Region) %>%
  filter(n() > 3) %>%
  ungroup()

# make a summary with counts
# filter out of total_datapoints any region with 3 or fewer counts

region_weights_graph <- ggplot(total_datapoints_counts_filtered, aes(
                  x= reorder(Region, Coefficient_Weight), 
                  y= Coefficient_Weight, 
                  fill = seed_category, 
                  color = seed_category)) +
    coord_flip() +
 #   facet_wrap(~seed_category) +
    geom_boxplot(alpha = 0.5, lwd = 1, position = "dodge") +
    geom_point(alpha = 0.8, position = position_jitterdodge()) +
    geom_hline(yintercept = 0, linetype = "dashed") +   #hline because coord_flip
    scale_y_continuous(limits = c(-0.4,0.2), expand = c(0,0), breaks = c(-0.4, 0, 0.2)) +
    scale_fill_manual(name = "Seed category", 
                      values = c("#006666","#7573b6"), 
                      labels = c("Accurate", "Inaccurate")) +
    scale_color_manual(name = "Seed category",
                       values = c("#006666","#7573b6"),
                       labels = c("Accurate", "Inaccurate")) +
    ggtitle("Coefficient Weight") +
    labs(subtitle = "Bootstrapping on WT larvae\nFiltered >3 Instances") +
    xlab("Region") +
    ylab("Coefficient Weight") +
    labs(fill = "Seed Category") +
    theme(
      text=element_text(family="Arial"),
      panel.background = element_blank(),
      panel.spacing = unit(1, "lines"),
      axis.line = element_line(color = "grey40"),
      panel.grid.major.y = element_line(color = "grey80"),
      strip.text.x = element_text(size = 10)
  )

region_weights_graph
```

```
QRFP_weights_datapoints <- total_datapoints %>%
  filter(Region == "rhombencephalon_qrfp_neuron_cluster_sparse")

QRFP_weights_graph <- ggplot(QRFP_weights_datapoints, aes(
                  x= seed_category, 
                  y= Coefficient_Weight, 
                  fill = seed_category, 
                  color = seed_category)) +
    geom_boxplot(alpha = 0.5, lwd = 1, position = "dodge") +
    geom_point(size = 1, alpha = 0.8, position = position_jitterdodge()) +
    geom_hline(yintercept = 0, linetype = "dashed") +   #hline because coord_flip
    scale_y_continuous(limits = c(0,0.12), expand = c(0,0)) +
    scale_fill_manual(name = "Seed category", 
                      values = c("#006666","#7573b6"), 
                      labels = c("Accurate", "Inaccurate")) +
    scale_color_manual(name = "Seed category",
                       values = c("#006666","#7573b6"),
                       labels = c("Accurate", "Inaccurate")) +
    xlab("Region") +
    ylab("Coefficient Weight") +
    theme(
      text=element_text(family="Arial"),
      panel.background = element_blank(),
      panel.spacing = unit(1, "lines"),
      axis.line = element_line(color = "grey40"),
      panel.grid.major.y = element_line(color = "grey80"),
      axis.title = element_text(size = 10),
      axis.text = element_text(size = 8),
      strip.text.x = element_text(size = 8),
      legend.position = "none"
  )

#QRFP_weights_graph

# ggsave("RegionWeights_210806.png",       
#        device = "png",
#        width = 8, 
#        height = 5, 
#        units = ('in'),
#        dpi = 600)
# 
# ggsave("RegionWeights_210806.tif",
#       device = "tiff",
#        width = 8, 
#        height = 5, 
#        units = ('in'),
#        dpi = 600)
# 
# write_csv(total_datapoints_counts_filtered, "210809_RegionWeights_Datapoints_CountsFiltered.csv")
```

# 8 Miscellaneous other analyses

## 8.1 Evaluating correlations between the DCR6 and other individual brain regions

Note: for all correlational analyses, we will be using the full set of brains collected of all phenotypes and genotypes, even larvae with no known genotype and larvae with the “MID” phenotype that did not have sufficient n for phenotypic categorical analysis. This is appropriate because here we are examining patterns in the CaSR-EGFP expression data across brain regions, not analyzing based on phenotype or genotype.

### 8.1.1 Calculating highest VIF of the DCR6

The highest Pearson correlation of the Rhombencephalon QRFP neuron cluster is with Rhombencephalon Vglut2 Cluster 2. VIF here is ~5. VIF between 5 and 10 is a sign of “moderate collinearity” and VIF >10 is a dangerous degree of collinearity for a LASSO regression. So, the level of multicollinearity in our analysis was reasonable for a LASSO regression.

See also “Collinearity: a review of methods to deal with it and a simulation study evaluating their performance” by Dormann et al. (full citation in main manuscript).

```
# Making linear regression of signal in DCR6 vs signal in most correlated region, Rhombencephalon VGlut2 Cluster 2
RhQRFP_vs_RhVGlut2Cl2.lm = lm(rhombencephalon_qrfp_neuron_cluster_sparse ~ rhombencephalon_vglut2_cluster_2, data=normed)
 
max_r2 <- summary(RhQRFP_vs_RhVGlut2Cl2.lm)$r.squared
print(paste("Maximum R-squared of any brain region with DCR6 is", max_r2))
```

```
## [1] "Maximum R-squared of any brain region with DCR6 is 0.802330241352182"
```

```
max_vif <- 1/(1-max_r2)
print(paste("Maximum Variance Inflation Factor (VIF) of any brain region with DCR6 is", max_vif))
```

```
## [1] "Maximum Variance Inflation Factor (VIF) of any brain region with DCR6 is 5.05894278841949"
```

```
# Extract R squared and P value from linear model
RhQRFP_vs_VGlut2Cl2.r2 <- round(summary(RhQRFP_vs_RhVGlut2Cl2.lm)$r.squared, digits = 4)
RhQRFP_vs_VGlut2Cl2.p <- round(summary(RhQRFP_vs_RhVGlut2Cl2.lm)$coefficients[2,4], digits = 12)

# Write a caption for the graph -- note P value is rounding to 0 even with 12 digits, so I will just call it <0.0001
annotate_VGlut2Cl2 <- paste("R-squared =", RhQRFP_vs_VGlut2Cl2.r2, "; p < 0.0001")
 
ggplot(normed, aes(x = rhombencephalon_qrfp_neuron_cluster_sparse, y = rhombencephalon_vglut2_cluster_2)) +
   geom_point() +
   geom_smooth(method = "lm") +
     geom_point() +
  geom_smooth(method = "lm") +
  scale_y_continuous("CaSR-EGFP Signal in VGlut2 Cluster 2", 
                    # breaks = c(0,.25,.50,.75,1),
                     limits = c(0,12),
                     expand = c(0,0.1)) +
  scale_x_continuous("CaSR-EGFP Signal in the DCR6") +
  annotate("text", x = 10, y = 1, label = annotate_VGlut2Cl2) +
    theme(
        plot.title = element_text(size = 12, face = "bold", hjust = 0.5),
        axis.text.x = element_text(color = "black", size = 8, angle = 45, hjust = 1),
        axis.line = element_line(color = "black"),
       # axis.text.x = element_blank(),
        axis.text.y = element_text(color = "black", size = 8),
        panel.grid.major.y = element_blank(),
        panel.grid.major.x = element_blank(),
        panel.background = element_rect(fill = NA),
        #legend.position = "none",
        axis.title.y = element_text(size=10, face = "bold"),
        axis.title.x = element_text(size=10, face = "bold"),
        strip.text = element_text(size = 10))
```

### 8.1.2 Correlation between DCR6 and hypothalamic QRFP+ cluster

The QRFP:GFP line labels the DCR6 sparsely, hence its anatomical database name of “Rhombencephalon QRFP cluster - sparse.” However, only the hypothalamic QRFP cluster is labeled by a QRFP RNA *in situ* (see Chen et al. 2016). There are a few possible explanations for this discrepancy:

- The DCR6 might contain projections from the hypothalamic QRFP cluster
- The DCR6 and the hypothalamic QRFP+ cluster might have a common progenitor population
- The DCR6 might be labeled by a genomic enhancer near the integration site of the QRFP:GFP transgenic construct

If the second scenario is true, we might expect the DCR6 and the hypothalamic QRFP cluster to be highly correlated in their CaSR-EGFP signal levels, since presumably epigenetic silencing of the CaSR-EGFP construct is occurring throughout development (otherwise, it is hard to account for the diverse patterns of silencing in larvae from the same clutches).

However, analysis suggests signal in these regions is not especially highly correlated for this dataset (R-squared is low).

```
# Make a linear model
RhQRFP_vs_HyQRFP.lm = lm(rhombencephalon_qrfp_neuron_cluster_sparse ~ diencephalon_hypothalamus_qrfp_neuron_cluster, data=normed)
 
# Extract the R-squared and p value from the linear model
RhQRFP_vs_HyQRFP.r2 <- round(summary(RhQRFP_vs_HyQRFP.lm)$r.squared, digits = 2)
RhQRFP_vs_HyQRFP.p <- round(summary(RhQRFP_vs_HyQRFP.lm)$coefficients[2,4], digits = 2)

# Write a caption for the graph
annotate_HyQRFP <- paste("R-squared =", RhQRFP_vs_HyQRFP.r2, "; p =", RhQRFP_vs_HyQRFP.p)

# Make graph
DCR6_vs_hypothal_QRFP <- ggplot(normed, 
                                aes(x = rhombencephalon_qrfp_neuron_cluster_sparse, 
                                    y = diencephalon_hypothalamus_qrfp_neuron_cluster)) +
  geom_point() +
  geom_smooth(method = "lm") +
  scale_y_continuous("CaSR-EGFP Signal in Hypothalamus QRFP+ Region", 
                    # breaks = c(0,.25,.50,.75,1),
                     limits = c(0,2),
                     expand = c(0,0.1)) +
  scale_x_continuous("CaSR-EGFP Signal in the DCR6") +
  annotate("text", x = 10, y = 1, label = annotate_HyQRFP) +
    theme(
        plot.title = element_text(size = 12, face = "bold", hjust = 0.5),
        axis.text.x = element_text(color = "black", size = 8, angle = 45, hjust = 1),
        axis.line = element_line(color = "black"),
       # axis.text.x = element_blank(),
        axis.text.y = element_text(color = "black", size = 8),
        panel.grid.major.y = element_blank(),
        panel.grid.major.x = element_blank(),
        panel.background = element_rect(fill = NA),
        #legend.position = "none",
        axis.title.y = element_text(size=10, face = "bold"),
        axis.title.x = element_text(size=10, face = "bold"),
        strip.text = element_text(size = 10)) 

# Display graph
DCR6_vs_hypothal_QRFP
```

### 8.1.3 DCR6 versus trigeminal ganglion

To validate the role that the DCR6 plays in decision-making, we acquired two Gal4 lines that drive expression in the DCR6. However, they also drive expression in the trigeminal and vagal ganglia. We wanted to investigate if there was also a high correlation between CaSR expression in the DCR6 and in the trigeminal and vagal ganglia in our pan-neuronal variegated expression data.

Our first analysis revealed that CaSR-EGFP almost never expressed in the trigeminal ganglion when driven by alpha tubulin Gal4. R-squared for the correlation is low.

```
# Make a linear model
RhQRFP_vs_TrigG.lm = lm(rhombencephalon_qrfp_neuron_cluster_sparse ~ ganglia_trigeminal_ganglion, data=normed)
 
# Extract the R-squared and p value from the linear model
RhQRFP_vs_TrigG.r2 <- round(summary(RhQRFP_vs_TrigG.lm)$r.squared, digits = 2)
RhQRFP_vs_TrigG.p <- round(summary(RhQRFP_vs_TrigG.lm)$coefficients[2,4], digits = 2)

# Write a caption for the graph
annotate_TrigG <- paste("R-squared =", RhQRFP_vs_TrigG.r2, "; p =", RhQRFP_vs_TrigG.p)

# Make graph
DCR6_vs_trig_gang <- ggplot(normed, 
                                aes(x = rhombencephalon_qrfp_neuron_cluster_sparse, 
                                    y = ganglia_trigeminal_ganglion)) +
  geom_point() +
  geom_smooth(method = "lm") +
  scale_y_continuous("CaSR-EGFP Signal in Trigeminal Ganglion", 
                    # breaks = c(0,.25,.50,.75,1),
                     limits = c(0,2),
                     expand = c(0,0.1)) +
  scale_x_continuous("CaSR-EGFP Signal in the DCR6") +
  annotate("text", x = 10, y = 1, label = annotate_TrigG) +
    theme(
        plot.title = element_text(size = 12, face = "bold", hjust = 0.5),
        axis.text.x = element_text(color = "black", size = 8, angle = 45, hjust = 1),
        axis.line = element_line(color = "black"),
       # axis.text.x = element_blank(),
        axis.text.y = element_text(color = "black", size = 8),
        panel.grid.major.y = element_blank(),
        panel.grid.major.x = element_blank(),
        panel.background = element_rect(fill = NA),
        #legend.position = "none",
        axis.title.y = element_text(size=10, face = "bold"),
        axis.title.x = element_text(size=10, face = "bold"),
        strip.text = element_text(size = 10)) 

# Display graph
DCR6_vs_trig_gang
```

### 8.1.4 DCR6 versus vagal ganglion

Next, we investigated the vagal ganglion and likewise found a low R-squared, indicating weak correlation between the DCR6 and this area.

```
# Make a linear model
RhQRFP_vs_VagG.lm = lm(rhombencephalon_qrfp_neuron_cluster_sparse ~ ganglia_vagal_ganglia, data=normed)
 
# Extract the R-squared and p value from the linear model
RhQRFP_vs_VagG.r2 <- round(summary(RhQRFP_vs_VagG.lm)$r.squared, digits = 2)
RhQRFP_vs_VagG.p <- round(summary(RhQRFP_vs_VagG.lm)$coefficients[2,4], digits = 4)

# Write a caption for the graph
annotate_VagG <- paste("R-squared =", RhQRFP_vs_VagG.r2, "; p =", RhQRFP_vs_VagG.p)

# Make graph
DCR6_vs_vag_gang <- ggplot(normed, 
                                aes(x = rhombencephalon_qrfp_neuron_cluster_sparse, 
                                    y = ganglia_vagal_ganglia)) +
  geom_point() +
  geom_smooth(method = "lm") +
  scale_y_continuous("CaSR-EGFP Signal in Vagal Ganglion", 
                    # breaks = c(0,.25,.50,.75,1),
                     limits = c(0,2),
                     expand = c(0,0.1)) +
  scale_x_continuous("CaSR-EGFP Signal in the DCR6") +
  annotate("text", x = 10, y = 1, label = annotate_VagG) +
    theme(
        plot.title = element_text(size = 12, face = "bold", hjust = 0.5),
        axis.text.x = element_text(color = "black", size = 8, angle = 45, hjust = 1),
        axis.line = element_line(color = "black"),
       # axis.text.x = element_blank(),
        axis.text.y = element_text(color = "black", size = 8),
        panel.grid.major.y = element_blank(),
        panel.grid.major.x = element_blank(),
        panel.background = element_rect(fill = NA),
        #legend.position = "none",
        axis.title.y = element_text(size=10, face = "bold"),
        axis.title.x = element_text(size=10, face = "bold"),
        strip.text = element_text(size = 10)) 

# Display graph
DCR6_vs_vag_gang
```

# 9 Session Information

```
sessionInfo()
```

```
## R version 4.0.5 (2021-03-31)
## Platform: x86_64-w64-mingw32/x64 (64-bit)
## Running under: Windows 10 x64 (build 19043)
## 
## Matrix products: default
## 
## locale:
## [1] LC_COLLATE=English_United States.1252 
## [2] LC_CTYPE=English_United States.1252   
## [3] LC_MONETARY=English_United States.1252
## [4] LC_NUMERIC=C                          
## [5] LC_TIME=English_United States.1252    
## 
## attached base packages:
## [1] grid      stats     graphics  grDevices datasets  utils     methods  
## [8] base     
## 
## other attached packages:
##  [1] renv_0.15.2          corrplot_0.90        circlize_0.4.13     
##  [4] ComplexHeatmap_2.4.3 gt_0.3.1             here_1.0.1          
##  [7] ggpubr_0.4.0         hablar_0.3.0         janitor_2.1.0       
## [10] readxl_1.3.1         glmnet_4.1-3         Matrix_1.3-2        
## [13] forcats_0.5.1        stringr_1.4.0        dplyr_1.0.7         
## [16] purrr_0.3.4          readr_2.0.2          tidyr_1.1.4         
## [19] tibble_3.1.5         ggplot2_3.3.5        tidyverse_1.3.1     
## 
## loaded via a namespace (and not attached):
##  [1] colorspace_2.0-2    ggsignif_0.6.3      rjson_0.2.21       
##  [4] ellipsis_0.3.2      rio_0.5.27          rprojroot_2.0.2    
##  [7] snakecase_0.11.0    GlobalOptions_0.1.2 fs_1.5.0           
## [10] clue_0.3-60         rstudioapi_0.13     farver_2.1.0       
## [13] bit64_4.0.5         fansi_0.5.0         lubridate_1.8.0    
## [16] xml2_1.3.2          codetools_0.2-18    splines_4.0.5      
## [19] knitr_1.36          jsonlite_1.7.2      broom_0.7.10       
## [22] cluster_2.1.2       dbplyr_2.1.1        png_0.1-7          
## [25] BiocManager_1.30.16 compiler_4.0.5      httr_1.4.2         
## [28] backports_1.3.0     assertthat_0.2.1    fastmap_1.1.0      
## [31] cli_3.1.0           htmltools_0.5.2     tools_4.0.5        
## [34] gtable_0.3.0        glue_1.4.2          Rcpp_1.0.7         
## [37] carData_3.0-4       cellranger_1.1.0    jquerylib_0.1.4    
## [40] vctrs_0.3.8         nlme_3.1-152        iterators_1.0.13   
## [43] xfun_0.27           openxlsx_4.2.4      rvest_1.0.2        
## [46] lifecycle_1.0.1     rstatix_0.7.0       scales_1.1.1       
## [49] vroom_1.5.5         hms_1.1.1           parallel_4.0.5     
## [52] RColorBrewer_1.1-2  yaml_2.2.1          curl_4.3.2         
## [55] sass_0.4.0          stringi_1.7.5       highr_0.9          
## [58] foreach_1.5.1       checkmate_2.0.0     zip_2.2.0          
## [61] shape_1.4.6         rlang_0.4.12        pkgconfig_2.0.3    
## [64] evaluate_0.14       lattice_0.20-41     labeling_0.4.2     
## [67] bit_4.0.4           tidyselect_1.1.1    magrittr_2.0.1     
## [70] R6_2.5.1            generics_0.1.1      DBI_1.1.1          
## [73] mgcv_1.8-34         pillar_1.6.4        haven_2.4.3        
## [76] foreign_0.8-81      withr_2.4.2         survival_3.2-10    
## [79] abind_1.4-7         modelr_0.1.8        crayon_1.4.2       
## [82] car_3.0-11          utf8_1.2.2          tzdb_0.2.0         
## [85] rmarkdown_2.11      GetoptLong_1.0.5    data.table_1.14.2  
## [88] reprex_2.0.1        digest_0.6.28       munsell_0.5.0
```
