# Supplementary material for: Multivariate analysis of variegated expression in Neurons: A strategy for unbiased localization of gene function to candidate brain regions in larval zebrafish
Source: PLoS One. 2023 Feb 14;18(2):e0281609. doi: 10.1371/journal.pone.0281609 (PMC9928119; doi:10.1371/journal.pone.0281609)
Supplement: Attachment — Submitted filename: Response to Reviewers.docx [file pone.0281609.s004.docx]

**Response to Reviewers**

We appreciate the reviewer for their time and thoughtful comments on our manuscript. The reviewer requests clarification regarding our use of the term “gain-of-function” phenotype as opposed to “overexpression phenotype.” We agree that our initial choice of language was potentially confusing. According to the reviewer’s suggestion, we have changed the language throughout the manuscript to reflect that we performed our analysis on larvae that overexpressed CaSR in neurons in a variegated fashion, and that this overexpression produced a behavioral phenotype. We now avoid the term “gain-of-function” to avoid potential confusion with gain-of-function mutations, which we did not employ in this manuscript. We thank the reviewer for their helpful suggestion.
